# Unravelling the Multiple Functions of the Architecturally Intricate *Streptococcus pneumoniae* β-galactosidase, BgaA

**DOI:** 10.1371/journal.ppat.1004364

**Published:** 2014-09-11

**Authors:** Anirudh K. Singh, Benjamin Pluvinage, Melanie A. Higgins, Ankur B. Dalia, Shireen A. Woodiga, Matthew Flynn, Audrey R. Lloyd, Jeffrey N. Weiser, Keith A. Stubbs, Alisdair B. Boraston, Samantha J. King

**Affiliations:** 1 Center for Microbial Pathogenesis, The Research Institute at Nationwide Children's Hospital, Columbus, Ohio, United States of America; 2 Biochemistry and Microbiology, University of Victoria, Victoria, British Columbia, Canada; 3 Department of Microbiology, University of Pennsylvania, Philadelphia, Pennsylvania, United States of America; 4 Department of Pediatrics, The Ohio State University, Columbus, Ohio, United States of America; 5 School of Biomedical, Biomolecular and Chemical Sciences, The University of Western Australia, Crawley, Western Australia, Australia; National Jewish Medical and Research Center, United States of America

## Abstract

Bacterial cell-surface proteins play integral roles in host-pathogen interactions. These proteins are often architecturally and functionally sophisticated and yet few studies of such proteins involved in host-pathogen interactions have defined the domains or modules required for specific functions. *Streptococcus pneumoniae* (pneumococcus), an opportunistic pathogen that is a leading cause of community acquired pneumonia, otitis media and bacteremia, is decorated with many complex surface proteins. These include β-galactosidase BgaA, which is specific for terminal galactose residues β-1–4 linked to glucose or N-acetylglucosamine and known to play a role in pneumococcal growth, resistance to opsonophagocytic killing, and adherence. This study defines the domains and modules of BgaA that are required for these distinct contributions to pneumococcal pathogenesis. Inhibitors of β-galactosidase activity reduced pneumococcal growth and increased opsonophagocytic killing in a BgaA dependent manner, indicating these functions require BgaA enzymatic activity. In contrast, inhibitors increased pneumococcal adherence suggesting that BgaA bound a substrate of the enzyme through a distinct module or domain. Extensive biochemical, structural and cell based studies revealed two newly identified non-enzymatic carbohydrate-binding modules (CBMs) mediate adherence to the host cell surface displayed lactose or N-acetyllactosamine. This finding is important to pneumococcal biology as it is the first adhesin-carbohydrate receptor pair identified, supporting the widely held belief that initial pneumococcal attachment is to a glycoconjugate. Perhaps more importantly, this is the first demonstration that a CBM within a carbohydrate-active enzyme can mediate adherence to host cells and thus this study identifies a new class of carbohydrate-binding adhesins and extends the paradigm of CBM function. As other bacterial species express surface-associated carbohydrate-active enzymes containing CBMs these findings have broad implications for bacterial adherence. Together, these data illustrate that comprehending the architectural sophistication of surface-attached proteins can increase our understanding of the different mechanisms by which these proteins can contribute to bacterial pathogenesis.

## Author Summary

The adherence of bacteria to host cells is a critical step in most bacterial infections; yet, mechanisms are poorly understood for many bacteria, including *Streptococcus pneumoniae* (pneumococcus), a human pathogen of global relevance. The surface of this bacterium is decorated with a landscape of large and structurally sophisticated proteins that mediate contact with the host. Here we show that the sugar-degrading β-galactosidase BgaA, can bind and cleave sugars through separate portions of this protein, which is one of the largest pneumococcal surface proteins and a model for architecturally intricate carbohydrate-active surface proteins. Non-enzymatic carbohydrate-binding modules in BgaA mediate adherence to specific host-cell surface carbohydrates. The identification of the first adhesin-carbohydrate receptor pair in *S. pneumoniae* provides critical molecular-level support for the long-held hypothesis that pneumococci bind carbohydrates on host cells and extends the paradigm of carbohydrate-binding module function. The enzymatically active portion of BgaA enables the bacterium to grow on host-derived glycans and evade the immune system, aspects of the host-pathogen interaction we show can be modulated by a specific inhibitor of enzymatic activity. Our work advances the concept that large bacterial surface proteins mediate complex host-bacterial interactions through specific functions of the varied regions comprising these proteins.

## Introduction

The cell surfaces of bacterial pathogens are complex landscapes of molecules that create an elaborate interface between the host and the bacterium. Integral to this landscape are cell-surface presented proteins that provide a variety of functions from cellular maintenance to communicating with the external environment to interaction with host tissues. A common feature of these proteins, particularly in Gram-positive bacteria, is their very large size and structural sophistication. These architecturally intricate proteins are also often functionally complex and thereby contribute to different aspects of pathogenesis.

Carbohydrate-active enzymes (CAZymes), particularly those that break glycosidic bonds joining sugar residues, are frequently found on the surface of bacterial species and are commonly architecturally intricate. By definition these enzymes contain a catalytic domain that confers the ability to break glycosidic bonds; the most common super-family is the glycoside hydrolases (GH), which are further organized into families based on sequence similarity [1]. GHs often contain numerous ancillary modules, the most common of which are the carbohydrate-binding modules (CBM) that non-catalytically mediate enzyme-carbohydrate interactions [2], [3]. The paradigm of CBM function has been that these modules concentrate enzymes onto carbohydrate substrates and, through this local concentration effect, enhance catalytic activity. This, however, has been based largely on non-surface attached enzyme systems.

CAZymes have been a focus of study for the opportunistic pathogen *Streptococcus pneumoniae* (pneumococcus), a leading cause of pneumonia, bacteremia and meningitis. Pneumococci express eight known surface CAZymes that have distinct specificities and that together can modify a wide range of host glycans including N-linked glycans, O-linked glycans and glycosaminoglycans [4]–[6]. *S. pneumoniae* expresses several exoglycosidases that cleave terminal carbohydrates. Neuraminidase NanA cleaves terminal α-2,3 and α-2,6 linked sialic acid, while pneumococci express two β-galactosidases BgaA, specific for terminal galactose (Gal) β-1,4 linked to N-acetylglucosamine (GlcNAc) or glucose and BgaC, specific for terminal galactose β-1,3 linked to GlcNAc [7]–[9]. N-acetylglucosaminidase StrH, contains two GH20 catalytic modules that both recognize terminal GlcNAc residues that are β-1,2 linked to mannose within complex N-linked glycans, but have subtle differences in enzymatic activity [10], [11]. *S. pneumoniae* also expresses endoglycosidases EndoD, an Endo-β-N-acetylglucosaminidase, which cleaves the chitobiose core of N-linked glycans and Eng which cleaves the core-1 (Galβ-1,3 N-acetylgalactosamine) structure of O-linked glycans [12], [13]. *S. pneumoniae* also expresses α-glucanase SpuA, which has specificity for α-1,6 linkages of glucose in the context of stretches of α-1,4 linked glucose, such as those found in glycogen [14], [15]. Finally, pneumococci express a hyaluronate lyase (Hyl) that cleaves the β-1,4 linkage of hyaluronic acid by β-elimination [4], [16], [17]. Several of these enzymes contribute to the ability of pneumococci to colonize or cause disease *in vivo* [6], [18]–[21]. Furthermore, many of these CAZymes have been shown, using *in vitro* assays, to contribute to specific steps in pathogenesis including growth, avoidance of clearance by the immune system, adherence and biofilm formation [6], [15], [19], [20], [22]–[31].

**Figure 1:**
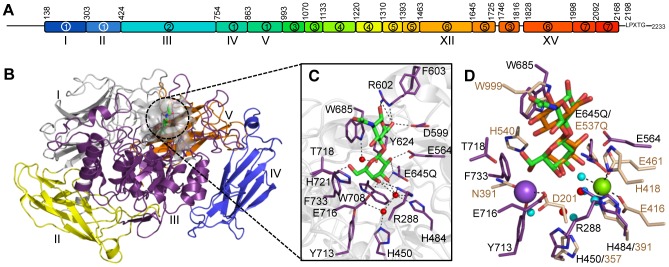
The structural features of BgaA from *S. pneumoniae*. (A) The architecture of BgaA showing the 17 modules/domains defined on the basis of fold recognition using the Phyre2 server [39]. The 7 different modules/domains are labeled with Arabic numbers: 1, sequence similarity to GH2-associated Ig-like; 2, sequence similarity to GH2 (β/α)_8_-barrel; 3, fold similarity to PDB ID 2LY7 (>98% confidence); 4, fold similarity to a fragment of a bacterial invasin (95% confidence); 5, fold similarity to bacterial Ig-like modules (98% confidence); 6, predicted β-sandwich fold similar to that of family 32 CBMs (99.5% confidence); 7, fold similarity to pneumococcal G5 modules (>98% confidence). The LPXTG cell wall anchoring motif is shown. The modules/domains that are the focus of this study are labeled beneath the schematic with Roman numerals. Amino acid numbering for the module/domain boundaries is given above the schematic. (B) Cartoon representation of the structure of the catalytic region comprising domains I–V (colored sequentially as gray, yellow, purple, blue, and orange). The bound LacNAc molecule is shown as green sticks and the surface of the active site in transparent gray. (C) Specific interactions of the BgaA active site with LacNAc (green). Water molecules are shown as red spheres and hydrogen bonds as dashed lines. (D) Overlap of the BgaA active site (purple stick representation for side chains, green sticks for LacNAc, and red spheres for waters) with the active site of *E. coli* LacZ in complex with lactose (tan stick representation for side chains, orange sticks for lactose, and blue spheres for waters, green sphere for Mg^2+^, and purple sphere for Na^+^; PDB ID 1JYN).

Pneumococcal surface-associated glycosidases are multimodular suggesting that they have complex interactions with soluble glyconjugates, mucin layers, and/or the glycocalyx layer that coats mammalian cells. At over 2200 amino acids and with at least 17 modules/domains of 7 different types the β-galactosidase BgaA, is among the largest cell surface attached proteins expressed by *S. pneumoniae* [7], [32] (Figure 1A). At present, none of the individual modules of BgaA have been ascribed functions and the functions of similar modules in other proteins, except the predicted catalytic module, remain ambiguous. The gene encoding BgaA is present in all sequenced pneumococcal strains and all strains tested possess β-galactosidase activity [32]–[35]. BgaA is specific for galactose β-1,4-linked to glucose or GlcNAc [lactose or N-acetyllactosamine (LacNAc) motifs, respectively] found in glycoconjugates. This activity is required for the release of galactose from N-linked glycans and for efficient growth on glycoconjugates having these modifications [5], [7], [22], [36], [37]. BgaA is also linked to pneumococcal resistance against complement deposition and the resulting phagocytic killing [23] and strongly involved with adherence to epithelial cells [31]. At present, deeper insight into the complex biological roles that BgaA plays is hindered by an absence of studies that relate the complex architecture of this enzyme to its varied functions.

In this study, the varied biological functions of BgaA are deconvoluted from the complex architecture of this enzyme. Through detailed structural and functional analyses the molecular basis for the catalytic specificity of BgaA is defined and this activity is demonstrated as critical for the ability of pneumococci to utilize complex N-linked glycans as a carbon source and protect the bacterium from opsonophagocytic killing. Further analyses also revealed the presence of non-catalytic CBMs within the C-terminal region of BgaA that mediate adherence to host cell surface LacNAc and/or lactose. Notably, this is the first demonstration that a CBM within a CAZyme can mediate adherence of a pathogen to host cells, thus extending the paradigm of CBM function. As CBM containing CAZymes are on the surface of many other bacterial species, we hypothesize that BgaA is a member of a novel class of adhesins. Furthermore, we show that these functions can be specifically modulated with small molecule inhibitors or competitors. Together these data highlight that understanding the architectural sophistication of surface-attached proteins can increase our understanding of the different mechanisms by which these proteins can contribute to bacterial pathogenesis and potentially aid in the development of strategies to inhibit these pathogenic mechanisms.

## Results

### Structure and inhibition of the BgaA catalytic region

The N-terminal region of BgaA comprising amino acid residues 138–993 has amino acid sequence identity with GH family 2 enzymes. The X-ray crystal structures of a catalytically active fragment of BgaA was determined to 2.7 Å resolution (data not shown) and an inactive Glu645Gln nucleophile mutant in complex with unhydrolyzed LacNAc to 2.2 Å resolution (Figure 1B and Figure S1A). This polypeptide had five distinct domains, four with immunoglobulin (Ig)-like folds that are arranged to create a nest in which the central (α/β)_8_-barrel domain III sits. All other structurally characterized GH2 enzymes with known β-galactosidase activity have a LacZ-type architecture where Ig-like domain V is replaced by a super β-sandwich domain (Figure S1B). The catalytic site of BgaA resides in a pocket located at the center of domain III (Figure 1B) and makes a series of direct and water-mediated hydrogen bonds with both residues of the disaccharide while the a-face of the GlcNAc residues lies parallel to Trp685 in a classical carbohydrate ring-aromatic amino acid sidechain interaction (Figure 1C). The LacNAc in this complex does not fully engage the catalytic residues: neither Glu564, the acid base, nor Gln645, mutated from the glutamic acid that would normally act as the nucleophile, are appropriately positioned to perform a catalytic function. This “shallow” mode of substrate binding representing an active site loading step is the same as that observed for *Escherichia coli* LacZ Glu537Gln mutant in complex with lactose (Figure 1D). The positions of the nucleophile and catalytic acid, Glu645 and Glu564, respectively, in BgaA are conserved with the analogous residues Glu537 and Glu461 of LacZ. Notably, however, there was no evidence of bound metals in the active site of BgaA. Indeed, the side chain of Arg288 occupies the space where a Mg^2+^ atom is bound in LacZ while Tyr713 and Glu716 fill the region occupied by a Na^+^ atom. Consistent with this, the activity of our catalytic region construct displayed no sensitivity to the presence or absence of metal ions (data not shown).

**Figure 2:**
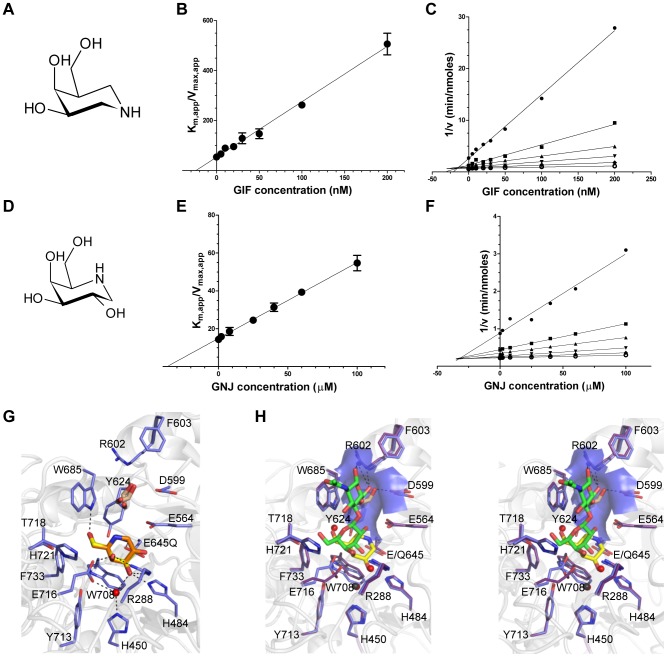
Characterization of BgaA inhibitors. (A) and (D) The chemical structures of GIF and GNJ, respectively. (B) and (E) Plots of the apparent Km against GIF and GNJ concentration, respectively. Solid lines represent the best fits from linear regression analysis. (C) and (F) Dixon plots of the data shown in panels (B) and (E) for GIF and GNJ, respectively. (G) Specific interactions of the BgaA active site with GIF (yellow sticks) and GNJ (orange sticks) with the protein from the GIF complex shown. Water molecules are shown as red spheres, ethylene glycol molecules as brown sticks, and hydrogen bonds as dashed lines. (H) Divergent stereo view of an overlap of the BgaA LacNAc complex (purple stick representation for side chains, green sticks for LacNAc, and red spheres for waters) with the BgaA GIF complex (blue stick representation for side chains, yellow sticks for LacNAc, brown sticks for a bound ethylene glycol, and black spheres for waters). The surface of the pocket accommodating the O6 of the GlcNAc residue is shown as transparent blue.

Given the shallow loading mode of LacNAc binding we also examined the binding of BgaA to the galactoisofagomine (GIF) and galactonojirimycin (GNJ), which are known potent galactosidase inhibitors [38] to provide additional insight into sugar recognition. GIF (Figure 2A) had a *K*_i_ of 25.0 (±4.4) nM (Figure 2B), displayed a competitive mode of inhibition (Figure 2C), and isothermal titration calorimetry (ITC; Figure S2A) confirmed the tight binding [*K*_d_ of 26.0 (±3.2) nM] and 1∶1 stoichiometry. GNJ (Figure 2D) had a more moderate *K*_i_ at 33.9 (±1.6) µM (Figure 2E) and also displayed a competitive mode of inhibition (Figure 2F). Despite the different chemical structures of the inhibitors they bound with very similar sets of interactions with Glu645 positioned beneath the atom equivalent to C1 at a distance of ∼3.5 Å, consistent with the role of this residue as a nucleophile (Figure 2G). GIF binding results in only subtle structural changes compared with LacNAc binding, despite the deeper binding mode of GIF (Figure 2H). These two complexes appear to represent a trajectory that progresses through a substrate-loading mode to a mode where the −1 catalytic subsite is fully engaged. The catalytically non-productive loading mode appears to provide BgaA with its substrate specificity through a pre-(−1)-subsite that recognizes terminal galactose residues and a preceding pre-(+1)-subsite that accommodates the β-1,4-linked GlcNAc residue through primary interactions with Trp685 and a series of hydrogen bonds between O6 of this sugar residue and a tailored pocket (Figure 2H). The steric constraints imposed by this architecture legislate against β-1,6-linked GlcNAc, with its longer overall length, and β-1,3-linked GlcNAc, where the 2-acetamido group would clash with the O6-specific pocket in the active site. Indeed, BgaA has insignificant activity on these sugars. This substrate-loading mode does not, however, suggest a mechanism for discrimination between lactose and LacNAc, where the latter is preferred by a factor of ∼10-fold [37]. It is possible that additional specificity for the 2-acetamido group of the GlcNAc is provided in the transition from the loading mode to fully involving the catalytic site and formation of the Michaelis complex where a deeper binding mode and/or distortion of the substrate might result in the engagement of this chemical group.

### BgaA catalytic activity is required for efficient growth on glycoconjugates and immune evasion, but not adherence

**Figure 3:**
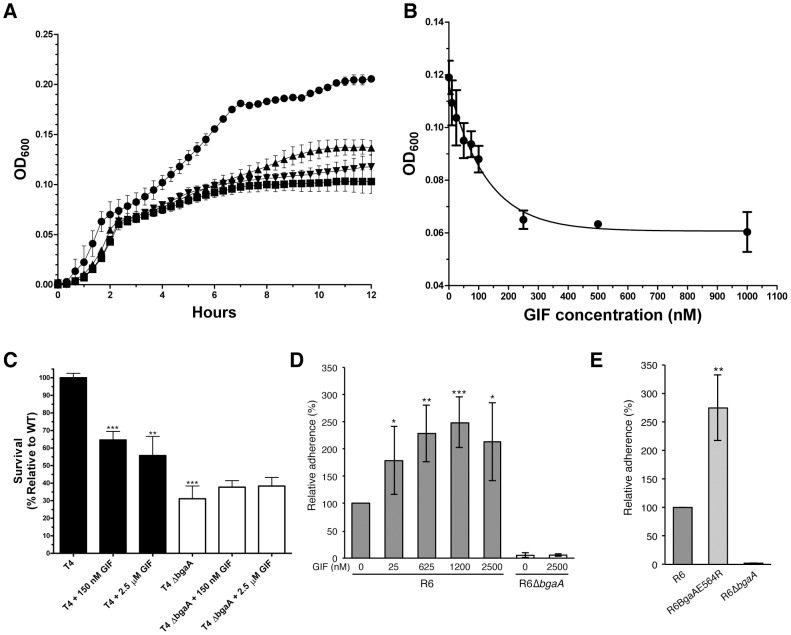
Inhibition of *S. pneumoniae* BgaA. (A) Growth curves of *S. pneumoniae* (TIGR4) performed using a semi-defined medium supplemented with bovine asialofetuin. Circles represent growth of the TIGR4 strain, triangles the growth of TIGR4 strain in the presence of 1000 nM GIF, and inverted triangles the growth of the Δ*bgaA* strain. Also shown as squares is the growth of the TIGR4 strain in the absence of a carbon source. Error bars represent the standard deviation of triplicate experiments run in parallel. The experiment was performed three times with highly similar results. (B) Inhibition of growth on asialofetuin as a function of GIF concentration. Results represent the mean measurements of three independent experiments where culture densities were taken at 6 hr of culture growth. The error bars represent the standard deviations of the independent measurements. (C) Survival of *S. pneumoniae* TIGR4 in neutrophil killing assays, showing comparisons of wild-type (filled bars) and Δ*bgaA* strain (open bars) in the presence and absence of GIF. Asterisks above sample bars represent statistical comparison of that sample with the reference, which is the TIGR4 strain with no inhibitor. Data are mean values compiled from two independent experiments performed in duplicate ± standard deviation. The Δ*bgaA* samples with inhibitors were compared with Δ*bgaA* in the absence of inhibitors and were found to have p values>0.1 and thus were not significantly different. (D) Addition of GIF (25–2500 nM) significantly increases adherence of *S. pneumoniae* R6 to D562 cells. Data are the mean ± SD of four independent experiments performed in triplicate. Asterisks above sample bars represent statistical comparison of R6 and R6 + GIF. (E) Adherence of an *S. pneumoniae* strain expressing enzymatically inactive BgaA (R6BgaAE564R) to D562 cells is significantly higher than the adherence of parental strain (R6). Data are the mean ± SD of three independent experiments performed in triplicate. Asterisks above sample bars represent statistical comparison between R6 and R6BgaAE564R. Statistically significant differences were assessed using a two-tailed Student's *t*-tests. * p≤0.05, ** p≤0.007, *** p≤0.0007.

As previously observed, deletion of *bgaA* resulted in significantly reduced growth on N-linked glycans decorating glycoproteins [22] (Figure 3A). The addition of 1 µM GIF reduced the growth of TIGR4 on aisalofetuin to approximately that of the *bgaA* mutant (Figure 3A). The reduction of growth by GIF was dose dependent with an inhibitor concentration ∼75 nM giving half the maximum reduction in growth, which is consistent with the measured *K*_i_ and *K*_d_ values (Figure 3B).

As previously reported, the survival of the *bgaA* mutant in an opsonophagocytic killing assay was reduced to ∼30% of that of the parental strain (Figure 3C) [23]. The addition of 150 nM or 2.5 µM GIF to the assays significantly reduced the survival of TIGR4 to ∼60%; GIF had no significant influence on the survival of the *bgaA* mutant (Figure 3C).

It has previously been reported that *bgaA* mutants in some genetic backgrounds including R6, but not TIGR4, were significantly reduced in adherence [31]. Consistent with the published data we observed a significant reduction in adherence of an R6 *bgaA* mutant to epithelial cells (Figure 3D). GIF did not reduce adherence of the R6 strain and indeed caused a significant increase in adherence in a dose dependent manner (Figure 3D). The concentration of GIF giving an approximately 50% increase in adherence was ∼25 nM, again consistent with the *K*_i_ determined for this inhibitor. GIF treatment resulted in a decrease in β-galactosidase activity associated with the bacterium indicating effective inhibition of BgaA catalytic activity (Figure S2D). This observation of increased adherence by inactivation of BgaA β-galactosidase activity was further supported by a similar increase in adherence of a mutant where substitution of the catalytic acid base residue, Glu564, by a bulky arginine residue to block the −1 subsite destroyed the catalytic activity of the enzyme (Figure 3E and Figure S2E). Thus, BgaA requires neither β-galactosidase activity nor an accessible active site to mediate adherence. Remarkably, the catalytic activity is in fact antagonistic to adherence.

Together these results show that the catalytic activity of BgaA is required for nutrient acquisition by this enzyme and protection from the innate immune system. Furthermore, these biological roles can be specifically inhibited by targeting the catalytic activity with an inhibitor. The mechanism by which the catalytic activity of BgaA provides protection from complement-mediated killing is presently unknown; however, it appears to be related to an effect of glycan modification, likely on complement components, that reduces complement deposition. In contrast, the catalytic activity of BgaA appears to inhibit adherence, suggesting that the portion of BgaA that mediates adherence is distinct from the catalytic site and, further, that the receptor may be a substrate for the BgaA catalytic region, and therefore a carbohydrate.

### The C-terminal region of BgaA is sufficient to facilitate pneumococcal adherence

**Figure 4:**
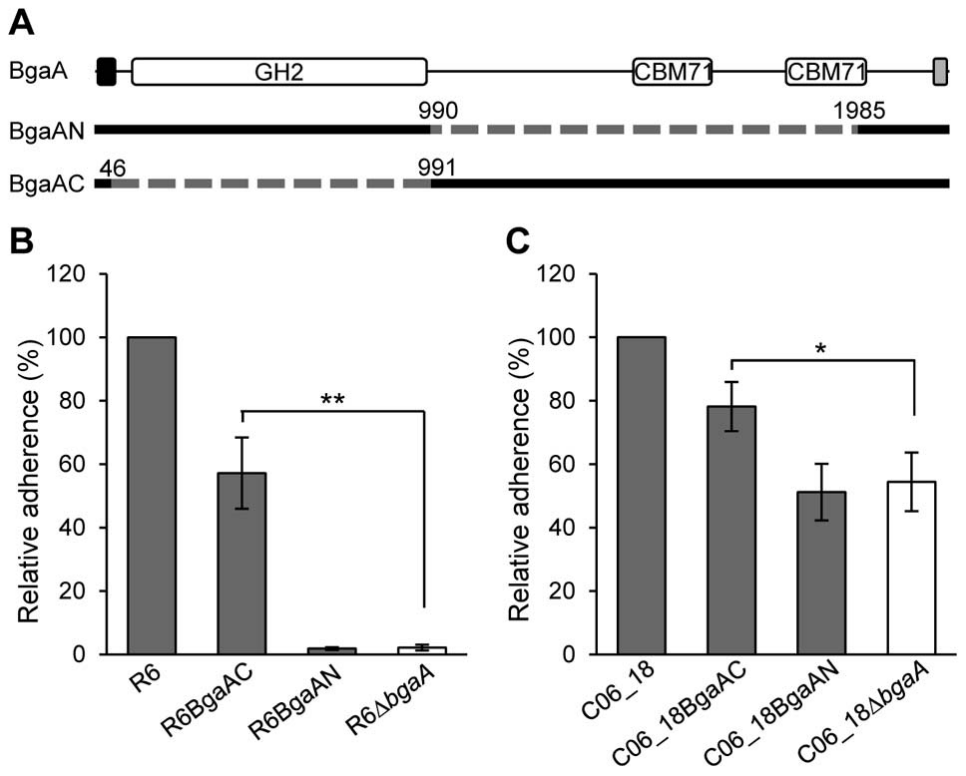
The C-terminal region of BgaA is sufficient to facilitate BgaA mediated pneumococcal adherence. (A) Schematic indicating regions of BgaA deleted to generate BgaAN and BgaAC. Dashed lines indicate regions deleted and numbers indicate amino acid boundaries of the deletions. The signal sequence, shown as the black box, and the Gram positive anchor domain, shown as a grey boxβ, were retained in both constructs for proper localization of expressed proteins. (B) R6BgaAC has significantly higher adherence to NHBE cells as compared to R6Δ*bgaA*. Adherence of R6BgaAN is not significantly different when compared to R6Δ*bgaA*. (C) CO6_18BgaAC has significantly higher adherence to NHBE cells as compared to CO6_18Δ*bgaA*. Adherence of CO6_18BgaAN is not significantly different when compared to CO6_18Δ*bgaA*. Adherence data are the means ± SD of three independent experiments performed in triplicate. Statistically significant differences were assessed using a two-tailed Student's *t*-tests. * p≤0.03, ** p≤0.002.

To test the hypothesis that the C-terminal region of BgaA mediates adherence, pneumococcal strains expressing either a surface-associated BgaA C-terminal region (BgaAC) or a surface-associated BgaA N-terminal enzymatic module (BgaAN) were constructed in strains previously used to demonstrate a role for BgaA in adherence, R6 and a low passage clinical isolate C06_18 (Figure 4A). For both strain backgrounds significantly higher adherence of the BgaAC strain as compared to the *bgaA* mutant to normal human bronchial epithelial (NHBE) cells and the pharyngeal cell line Detroit 562 (D562) was observed (Figures 4B, 4C, S3A and S3Bcbm). In contrast, BgaAN strains showed no significant difference in adherence from that of the *bgaA* mutant. An immunoblot was used to confirm that the N-terminal construct was properly expressed and localized (Figure S3C). Despite appropriate expression and localization, R6BgaAN had reduced β-galactosidase activity (Figure S3D); however, reduced adherence of R6BgaAN could not be attributed to reduced enzyme activity as catalytically inactive BgaA still facilitates efficient adherence (Figure 3E) [31].

The significant increase in adherence previously discerned in the absence of BgaA enzymatic activity was not observed for pneumococci expressing only the C-terminal region of BgaA, while the reason for this is unclear it may be that the large deletion affects surface presentation or stability of the protein. Nevertheless, these data indicate that the C-terminal region of BgaA mediates adherence to receptors on the epithelial cell surface. Furthermore, the observation that the catalytic activity of BgaA is antagonistic to adherence suggests that the receptor for BgaA adherence is the carbohydrate substrate of the catalytic domain.

### The C-terminal region of BgaA contains two CBMs that bind lactose and LacNAc

Amino acid sequence similarity searches failed to identify candidate CBMs in the C-terminal portion of BgaA. However, fold prediction using the Phyre2 server [39], which does not rely on amino acid sequence similarity, distinguished two regions (XII and XV, Figure 1A) with a high probability of adopting the β-sandwich fold common to many CBMs found in CAZymes. These two ∼175 amino acid residue modules, which we refer to as CBM71-1 and CBM71-2, share ∼35% amino acid identity with one another, but have no identity with known CBMs. The two predicted CBMs were recombinantly produced and the polypeptides screened for binding to all commonly occurring monosaccharides by UV difference spectroscopy; only D-galactose gave a signature UV difference spectrum consistent with sugar binding (Figures S4A and S4B). Subsequently, this approach was expanded to the relevant galactose-containing sugars LacNAc, lactose, galactopyranosyl-β-1,3-N-acetyl-D-glucosamine (lacto-N-biose), and galactopyranosyl-β-1,3-N-acetyl-D-galactosamine [Thomsen-Freidenreich (TF) epitope] and binding was only observed to LacNAc and lactose. For both CBMs, the binding to galactose was too weak to quantify. The dissociation constants (*K*_d_s) determined for CBM71-1 by ITC were 251 (±29) µM and 368 (±52) µM for LacNAc and lactose, respectively (Figures S4C and S4D). Similar values of 247 (±37) µM and 378 (±30) µM for LacNAc and lactose, respectively, were obtained for CBM71-2 (Figures S4E and S4F). Significantly, the CBMs only bound with significant affinity to sugars that are substrates for the catalytic domain. Though relatively weak, these affinities are consistent with those determined for other CBMs with similar binding specificities [40].

**Figure 5:**
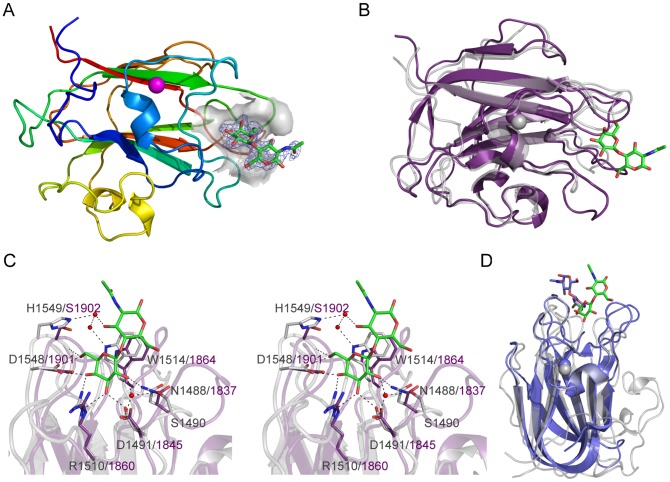
Structures of the CBM71 modules in BgaA. (A) A cartoon representation of CBM71-1 in complex with LacNAc. The protein is color ramped red to blue from the N-terminus to the C-terminus. A bound calcium atom is shown in magenta. The binding site of the CBM is shown as a grey transparent surface with the bound LacNAc molecule shown as green stick. The electron density for the LacNAc is shown as a blue-mesh *F*_o_-*F*_c_ maximum-likelihood/σ_A_-weighted map contoured at 3σ (0.33 e^−^/Å^3^). (B) An overlap of the structure of CBM71-2 (purple) with CBM71-1 (grey). The LacNAc molecule bound to CBM71-1 is shown as green sticks. (C) Expanded view of the CBM71 binding site shown in divergent stereo. CBM71-1 is shown in grey with the LacNAc shown as green sticks, residues involved in binding the LacNAc as grey sticks, and the interacting water network as red spheres. Black dashed lines represent potential hydrogen bonds. CBM71-2 is shown in purple; residues conserved with CBM71-2 are shown as purple sticks. (D) An overlap of the CBM71-1 LacNAc complex (grey with LacNAc in green) with the CBM32 from *C. perfringens* NagJ (blue with bound LacNAc shown as blue sticks).

The structure of CBM71-1 solved by X-ray crystallography in complex with LacNAc revealed its β-sandwich fold comprising opposing sheets of 4- and 5-anti-parallel β-strands (Figure 5A). A single bound metal ion was modeled as Ca^2+^ on the basis of coordination geometry and B-factor analysis. The shallow LacNAc binding site sits at the apex of the β-fold opposite the N- and C-termini (Figure 5A). The structure of CBM71-2 is highly similar to that of CBM71-1 with the most obvious difference being an extended loop adjacent to the binding site (Figure 5B). Though a bound complex of CBM71-2 was not obtained the binding sites of the two CBMs are very well conserved, consistent with the shared specificity of the CBMs and similar binding affinities (Figure 5C).

The base of the CBM71-1 active site provides amino acid sidechains that provide specificity for a terminal galacto-configured sugar but prevent accommodation of a 2-acetamido group, providing an explanation for the lack of binding to N-acetylgalactosamine (Figure 5C). Tryptophan 1514 lies directly beneath the glycoside bond and coplanar with the disaccharide thus providing CH-π interactions with both pyranose rings and a higher affinity for β-linked disaccharides than for galactose alone. This binding site architecture accommodates lactose and LacNAc, but would limit the recognition of other sugars terminating in β-linked galactose. Given their carbohydrate binding activity, but lack of amino acid sequence identity between the BgaA CBMs and known CBM families, CBM71-1 and CBM71-2 constitute the founding members of a new CBM family, CBM71, which is most similar in three-dimensional structure to CBM family 32 (Figure 5D).

### CBMs in BgaA mediate pneumococcal adherence

**Figure 6:**
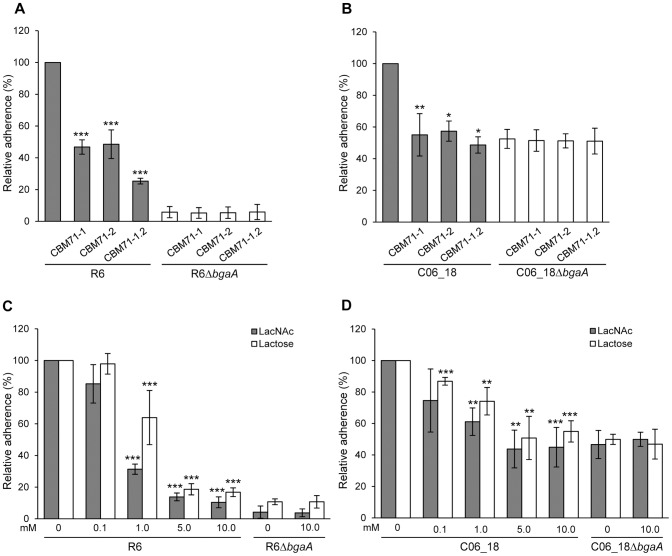
Recombinant CBMs and LacNAc and/or lactose reduce pneumococcal adherence in a BgaA-dependent manner. (A) Adherence of *S. pneumoniae* strain R6 and R6Δ*bgaA* to NHBE cells in the presence of CBM71-1, CBM71-2 or CBM71-1.2 (250 µM). Asterisks indicate significant differences in adherence in the presence or absence of CBM. (B) Adherence of *S. pneumoniae* strain C06_18 and C06_18Δ*bgaA* to NHBE cells in the presence of CBM71-1, CBM71-2 or CBM71-1.2 (250 µM). (C) Adherence of *S. pneumoniae* strain R6 and R6Δ*bgaA* to NHBE cells in the presence of LacNAc and lactose (0–10 mM). Asterisks indicate significant differences in adherence in the presence or absence of disaccharide. (D) Adherence of *S. pneumoniae* strain C06_18 and C06_18Δ*bgaA* to NHBE cells in the presence of LacNAc and lactose (0–10 mM). As in (C) except using pneumococcal strain C06_18. Adherence assays are mean ± SD of three independent experiments each performed in triplicate. Statistically significant differences were assessed using a two-tailed Student's *t*-test. * p≤0.04, ** p≤0.007, *** p≤8×10^−4^.

The ability of these CBMs to mediate adherence to host cells was explored using the free carbohydrates galactose, lactose and LacNAc as well as soluble recombinant CBMs as specific competitors of adherence. The addition of 250 µM CBM71-1 or CBM71-2 significantly reduced adherence of R6 and C06_18 to both NHBE and D562 cells (Figures 6A, 6B, S5A and S5B). The CBM71-1.2 tandem construct that comprises both CBMs and the two intervening modules reduced adherence more than either CBM alone; although, this difference was not significant for CO6_18. Importantly, recombinant CBMs had no significant effect on adherence of a *bgaA* mutant, demonstrating that the effect of CBMs on adherence was BgaA specific (Figures 6A, 6B, S5A and S5B).

Lactose, LacNAc and galactose significantly reduced adherence to NHBE and D562 cells, though, consistent with the low affinity of these CBMs for galactose, this monosaccharide reduced adherence (Figure S5E and data not shown) significantly less than the same concentration of disaccharides (Figure 6C, 6D, S5C and S5D). The effect of lactose and LacNAc was BgaA-specific and dose-dependent.

**Figure 7:**
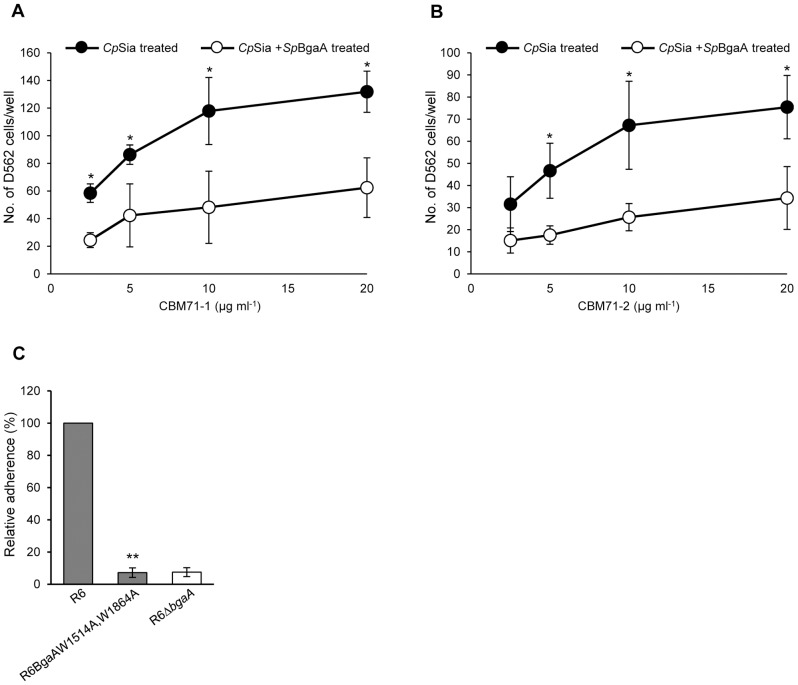
CBMs within BgaA directly bind epithelial cells. (A) Binding of D562 cells to immobilized recombinant CBM71-1. A range of concentrations (2.5–20 µg ml^−1^) of CBM71-1 were immobilized on the bottom of a 96 well plate. D562 cells pretreated with 0.002 Unit ml^−1^ *C. perfringens* sialidase (*Cp*Sia) with or without 0.054 µM *Sp*BgaA146-990 (*Sp*BgaA) were allowed to adhere to the plate. Adherence to control wells coated with 1 mg ml^−1^ of BSA was subtracted from the data (maximum 22 cells per well). Asterisks indicate significant differences in the number of adherent D562 cells following pretreatment with sialidase and sialidase plus *Sp*BgaA146-990. (B) Binding of D562 cells to immobilized recombinant CBM71-2. As in (A) except using CBM71-2. (C) Adherence of *S. pneumoniae* strain R6 and R6BgaAW1514A,W1864A to D562 cells. Asterisks indicate significant differences in adherence of R6 and R6BgaAW1514A,W1864A. Data are the mean ± SD of three independent experiments each performed in triplicate. Statistically significant differences were assessed using a two-tailed Student's *t*-test. * p≤0.04, ** p≤2.00×10^−7^.

Sialidase treated human epithelial cells showed significantly increased adherence to immobilized CBM71-1 and CBM71-2, as compared to immobilized BSA, indicating that the CBMs within BgaA directly interact with the host cell (Figure 7A and 7B). Furthermore, adherence to CBMs was reduced if epithelial cells were treated with both sialidase and the catalytic domain of BgaA, indicating that the receptor mediating adherence is a substrate of BgaA: terminal β-1,4-linked galactose. To ensure that this interaction was relevant in the context of intact bacteria we constructed a strain designed to abrogate CBM binding through point mutations in *bgaA* that target critical binding residues in the CBMs. W1514 and W1864 in the structures of CBM71-1 and CBM71-2, respectively, make classical aromatic amino acid side chain – carbohydrate ring interactions, which are typically critical to CBM binding [2]. Thus, these residues were chosen for alanine substitutions. As predicted, the strain expressing the *S. pneumoniae* mutant carrying the BgaAW1514A,W1864A variant showed dramatically reduced adherence that was not significantly different from the *bgaA* mutant (Figure 7C). This reduction in adherence was not due to differences in expression, localization or activity of BgaA (Figure S6A and S6B). Together these data strongly support the hypothesis that CBMs in BgaA contribute to pneumococcal adherence by binding to LacNAc and lactose containing cell surface glycoconjugates.

### Heterologous complementation of *S. pneumoniae bgaA* mutant by *Streptococcus gordonii bgaA*

**Figure 8:**
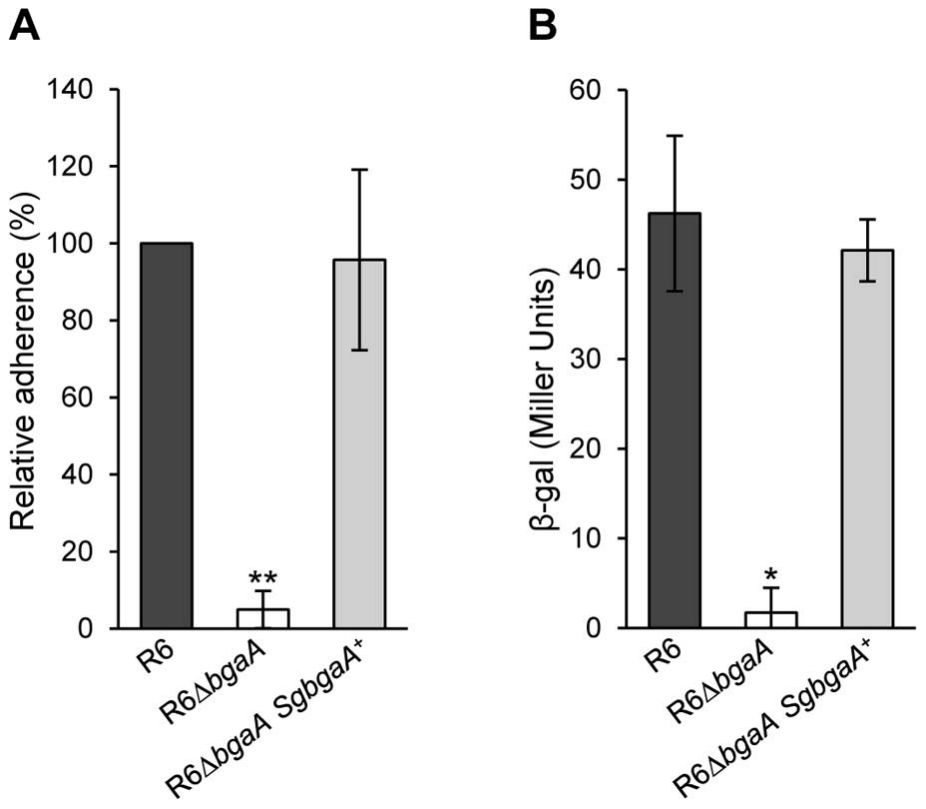
*S. gordonii bgaA* can restore the β-galactosidase activity and adherence of a *S. pneumoniae bgaA* mutant. (A) *S. gordonii bgaA* can restore the adherence of a *S. pneumoniae bgaA* mutant. (B) *S. gordonii bgaA* can restore the β-galactosidase activity of a *S. pneumoniae bgaA* mutant. Data presented here are mean ± SD of three independent experiments each performed in triplicate. Asterisks indicate statistically significant differences between R6Δ*bgaA* and both R6Δ*bgaA SgbgaA* ^+^ and R6 calculated using a two-tailed Student's *t*-test. * p≤0.002, ** p≤5×10^−6^.

Although the majority of β-galactosidases lack the large C-terminal region found within BgaA (Figure 4A), a relatively large number of host-adapted streptococci, including *S. gordonii*, encode similar β-galactosidases [41] (Figure S7). In order to test if BgaA orthologs may represent a previously uncharacterized class of bacterial adhesins, we tested adherence of an *S. pneumoniae bgaA* mutant expressing *S. gordonii* BgaA (R6Δ*bgaA SgbgaA* ^+^) at the same locus and under control of the native promoter. Adherence and enzymatic activity of the pneumococcal strain expressing the *S. gordonii* BgaA was not significantly different from that of the parental strain (Figure 8). These data indicate that other BgaA orthologs including *S. gordonii* BgaA have the potential to act as bacterial adhesins.

## Discussion

The catalytic specificity of BgaA is reported to be for LacNAc and lactose, carbohydrate motifs found on a wide variety of glycoconjugates, though the activity on lactose is lower [37]. This catalytic specificity appears to be initially provided by an unusual pre-active or substrate loading complex in the active site that is similar to what has been observed for *E. coli* LacZ and selects for the β-1,4-linkage in these sugars. The overall architecture of the active site, however, which generally accommodates only a disaccharide, suggests that the enzyme would be quite tolerant of sugar residues preceding a LacNAc or lactose motif, consistent with the ability of BgaA to release galactose from a wide variety of glycoconjugates terminating in LacNAc or lactose motifs [42]. Through the use of a *S. pneumoniae* mutant lacking *bgaA* the ability to process these sugar motifs has been linked to growth on a glycoconjugate and protection from opsonophagocytosis [5], [22], [23]. Here, the use of a tight binding inhibitor that specifically targets the active site of BgaA conclusively links the necessity of having an available catalytic site with these biological outcomes and indicates that glycan processing is responsible for the protective effect of BgaA against opsonophagocytosis.

We also identified ancillary CBMs that mediate adherence of the bacterium. Many bacterial species bind host tissues through protein-carbohydrate interactions, which is achieved through a potential myriad of proteins from single, dedicated surface proteins to components of complex flagellar structures [43]–[46]. This is, however, the first demonstration of a CBM mediating adherence of a pathogen to host cells. CBMs typically function to maintain CAZymes in proximity of substrate, thereby enhancing catalytic activity. This may indeed be also be the case with the CBMs in BgaA; however, the overall role in adhering the bacterium to a host cell is a new function for CBMs, not only expanding the repertoire of bacterial adhesins but altering the paradigm of CBM function. It may seem counterintuitive that adherence can be mediated by interactions of CBMs with host glycans that are cleaved by an enzymatic domain within the same protein. However, we propose a dynamic interaction between common host cell surface glycans and multiple copies of a bacterial surface protein. Multiple adhesion events also increase the avidity of the interaction and may provide an explanation of how CBMs with relatively weak affinity for glycans mediate adherence.

Though the CBMs in BgaA clearly mediate an interaction with carbohydrate motifs, namely LacNAc and lactose, the exact nature of the glycoconjugate receptor(s) remains unknown. LacNAc is very common in the N- and O-linked glycans that decorate glycproteins on the surface of epithelial cells while both LacNAc and lactose are frequent motifs in glycosphingolipids. BgaA is active on both LacNAc and lactose, albeit with approximately 10-fold higher activity on LacNAc [37], while the CBMs within BgaA show a minor preference for LacNAc, suggesting that LacNAc is the most likely receptor. Given that it has previously been reported that BgaA may bind a non-proteinacious receptor [31] the members of the neolactoceramide subfamily of glycosphingolipids, which contain LacNAc motifs, are possible candidates as glycoconjugate receptors for BgaA.

As other bacterial species adept at modifying carbohydrates encode surface-associated CAZymes predicted to contain CBMs [41], [47]–[49], we propose that BgaA may be a member of a novel class of bacterial adhesins. This hypothesis is supported by our data demonstrating that *bgaA* from *S. gordonii* can complement a *S. pneumoniae bgaA* mutant. In addition to BgaA, pneumococcal surface-associated glycoside hydrolases NanA, EndoD, Eng and SpuA, contain, or are predicted to contain, CBMs [1], [12], [50], [51]. Two of these enzymes, NanA and Eng, have been demonstrated to contribute to pneumococcal adherence [6], [26], [27]. Although NanA acts to reveal a receptor for BgaA-mediated adherence to epithelial cells, enzymatic activity is not required for adherence to endothelial cells [27]. In fact, an N-terminal region including a putative CBM is required for adherence to endothelial cells. The role of Eng in adherence remains to be defined.

It is likely that CBM-mediated adherence affects the pathogenesis of multiple bacterial species, but these data are of particular significance to the study of pneumococcal biology. It has long been proposed that initial adherence of pneumococci to host tissue occurs via binding carbohydrates on the epithelial cell surface but the identification of specific adhesin-receptor pairs has been lacking [52], [53]. This study elucidates the first carbohydrate-mediated pneumococcal adherence mechanism. It was previously reported that mutation of BgaA does not reduce adherence of all pneumococcal strains, but this mechanism of adherence is very likely relevant to pneumococcal pathogenesis as it affects adherence of multiple strains, including low-passage clinical isolates to all human airway epithelial cell lines tested and primary airway epithelial cells [31]. Pneumococci are a very diverse species and variances in the contribution of different adherence mechanisms between strains has previously been reported [54]–[56]. Differential expression of the five CBM containing CAZymes encoded by pneumococci could explain the differential role of BgaA to adherence, especially in light of the published evidence that some of these CAZymes contribute to adherence [6], [27].

Understanding the specific contributions of different domains/modules of complex proteins to bacterial pathogenesis provides the opportunity to identify inhibitors of these mechanisms. We significantly reduced pneumococcal adherence by the addition of recombinant CBM or free carbohydrate-receptor (i.e. lactose or LacNAc). Additionally, the tight binding β-galactosidase inhibitor GIF inhibited enzymatic activity on the surface of the bacteria to reduce growth on glycoconjugates and resistance to opsonophagocytosis. The demonstrated capacity to modulate the multiple functions of architecturally complex bacterial surface-associated CAZymes with simple molecules may provide a framework for developing approaches to targeting pathogens utilizing such proteins in the host-pathogen interaction.

## Materials and Methods

### Bacterial strains, plasmids and culture media

Bacterial strains and plasmids used in this study are described in Table S1. *S. pneumoniae S. gordonii*, and *E. coli* strains were grown using routine conditions for these organisms and where appropriate media was supplemented with antibiotics. For details see Supporting Information.

### Cloning, protein expression and purification

All cloning was performed using standard molecular biology procedures. Protein production in *E. coli* was done using pET 28-based expression vectors and purification of the polypeptides using procedures described previously and primers detailed in Table S2 [10]. Protein concentrations were determined by measuring the absorbance at 280 nm and using calculated molar extinction coefficient of 174070 cm^−1^.M^−1^ for GH2 and GH2-E645Q, 29540 cm^−1^.M^−1^ for CBM71-1, 33920 cm^−1^.M^−1^ for CBM71-2, and 76320 cm^−1^.M^−1^ for CBM71-1.2, [57]. For details see Supporting Information.

### Crystallography procedures

All crystallization experiments were performed using sitting-drop vapor diffusion for screening and hanging drop vapor diffusion for optimization, all at 18°C. Diffraction data were collected on cryo-protected crystals at 100 K and data was processed using MOSFLM and SCALA [58], [59]. All data collection and processing statistics are shown in Table S3. The structure of CBM71-1 was determined a by single-anomalous dispersion experiment optimized for selenium using the program ShelXC/D/E [60]. All other structures were solved by molecular replacement using standard procedures. For details see Supporting Information. All data collection, processing, and structure refinement statistics are given in Table S3.

### Enzyme inhibition and binding studies

All steady state kinetic studies were performed in triplicate at 37°C in a Cary/Varian 300 Bio UV-Visible Spectrophotometer as previously described [10]. The *K*_i_ values for GNJ and GIF were determined from plots of the apparent *K*_m_/V_max_ against inhibitor concentration. Qualitative UV difference scan and ITC were performed using methods already described [10], [40], [61], [62]. All experiments were performed at 25°C in triplicate. For details see Supporting Information.

### Generation of *S. pneumoniae* mutants

*S. pneumoniae* TIGR4Δ*bgaA* strain, was obtained by a PCR ligation technique to replace *bgaA* with a chloramphenicol cassette [15], [63]. *S. pneumoniae* R6 and C06_18 strains expressing the surface attached N-terminal (BgaAN) or C-terminal region of BgaA (BgaAC), R6 expressing enzymatically inactive BgaA (R6BgaAE564R), R6 expressing BgaA with point mutants in the CBMs that abrogate carbohydrate binding (R6BgaAW1514A,W1864A), and the *S. pneumoniae bgaA* mutant expressing *S. gordonii* BgaA (R6Δ*bgaA SgbgaA* ^+^) were generated using the Janus cassette selection method using primers described in Table S2 [64]. For details see Supporting Information.

### Growth assays and opsonophagocytic killing assays

The protocol for the growth assays of wild-type and Δ*bgaA S. pneumoniae* TIGR4 strains on bovine asialofetuin was adapted from Battig *et al.* [65] and performed as described previously [10]. Neutrophil killing assays were performed essentially as previously described with *S. pneumoniae* TIGR4 wild type strain with or without inhibitors and Δ*bgaA* strain in the presence of inhibitors or a vehicle control (+++ buffer) [10], [23], [66]. For details see Supporting Information.

### Adherence assays

Adherence *of S. pneumoniae* to monolayer of D562 cells (ATCC CCL-138) and primary NHBE cells (Lonza), grown in 24 well tissue culture plates was determined essentially as previously described [31], [67]. For details see Supporting Information.

### Cell adhesion assay to immobilized CBM

Ninety-six well plates coated in a range of concentrations of CBM71-1, CBM71-2 or BSA (control) were blocked with 1% BSA (w/v) before addition of D562 cells treated with *Clostridium perfringens* sialidase (*Cp*Sia) or sialidase and *S. pneumoniae* BgaA146-990 (*Sp*BgaA). Following incubation for 1 h at 37°C unbound cells were removed by washing and cells were fixed, stained and counted using an inverted light microscope. The average number of cells bound to BSA coated wells was subtracted from the number of cells attached to CBM coated wells. For details see Supporting Information.

### Statistical analysis

Data from opsonophagocytic, adherence assays and cell-binding assays were assessed for statistically significant differences using a two tailed Student's *t*-test and data points with *p* value≤0.05 were considered significant.

### Accession codes

Protein Data Bank. Coordinates and structure factors have been deposited with the following accession codes: native BgaA catalytic domain, 4cu6; BgaA catalytic domain in complex with GIF, 4cu7; BgaA catalytic domain in complex with GNJ, 4cu8; BgaA catalytic domain E645Q complex with LacNAc, 4cuc; CBM71-1 Se-met, 4cua; CBM71-1 in complex with LacNAc, 4cub; CBM71-2, 4cu9.

## Supporting Information

Figure S1 **Representative electron density for substrate bound to BgaA and comparison of** ***S. pneumoniae*** **BgaA with** ***E. coli*** **LacZ.** (A) LacNAc is represented in blue colored sticks with maximum likelihood/σ_A_ weighted 2*Fo-Fc* electron density map contoured at 0.26 electrons/Å^3^. (B) Cartoon representation of the structure of the BgaA catalytic region comprising domains I–V and colored sequentially as gray, yellow, purple, blue, and orange is overlapped with *E. coli* LacZ (tan).
(PDF)

Figure S2 **Inhibition of BgaA.** (A) Representative ITC titration for GIF titrated into the BgaA catalytic module. The solid line represents the best fit from non-linear regression analysis of using one-site binding model. (B) *S. pneumoniae* growth controls using a semi-defined medium with no carbon source. Circles represent growth of the TIGR4 strain supplemented with 1 µM purified BgaA catalytic domain, squares growth of the Δ*bgaA* strain supplemented with 1 µM purified BgaA catalytic domain, triangles the growth of TIGR4 strain, and inverted triangles the growth of the Δ*bgaA* strain. Error bars represent the standard deviation of triplicate experiments run in parallel. The experiment was performed multiple times with highly similar results. (C) *S. pneumoniae* growth controls using a semi-defined medium supplemented with bovine asialofetuin. Symbols are as above. (D) Activity of the cell-surface associated BgaA is significantly reduced in the presence of GIF (25–2500 nM). Data presented here are mean ± SD of three independent experiments each performed in triplicate. *Statistically significant reduction in β-galactosidase activity as compared to R6 in the absence of GIF (p≤0.0006). (E) Activity of R6BgaAE564R is significantly reduced as compared to the parental strain. Data presented here are mean ± SD of three independent experiments each performed in triplicate. *Statistically significant reduction in β-galactosidase activity (p≤0.0003) as compared to R6.
(PDF)

Figure S3 **Determining the role of N and C terminal regions of BgaA in pneumococcal adherence.** (A) R6BgaAC has significantly higher adherence to D562 cells as compared to R6Δ*bgaA*. Adherence of R6BgaAN is not significantly different when compared to R6Δ*bgaA*. (B) C06_18BgaAC has significantly higher adherence to D562 cells as compared to C06_18Δ*bgaA*. Adherence of C06_18BgaAN is not significantly different when compared to C06_18Δ*bgaA*. (C) The N-terminal enzymatic module of BgaA expressed by R6BgaAN is localized to the bacterial cell surface. Immunoblot of cytoplasmic (CP) and cell wall (CW) protein fractions for localization of full length BgaA expressed by parental strain (R6) and the BgaA N-terminal enzymatic module expressed by R6BgaAN. Deletion of BgaA amino acids 991–1984 in strain R6BgaAN does not alter the expression and localization of the protein. (D) R6BgaAN has reduced β-galactosidase activity compared to the parental strain; however, the level of activity is significantly higher than that of R6Δ*bgaA*. Data are the means ± SD of three independent experiments performed in triplicate. Statistically significant differences were assessed using a two-tailed Student's *t*-tests. * p≤0.03, ** p≤0.002.
(DOCX)

Figure S4 **Analysis of carbohydrate binding by the CBMs of BgaA using UV Difference spectroscopy and ITC.** (A) Example UV difference spectra of CBM71-1 caused by the presence of 1 mM LacNAc. (B) Example UV difference spectra of CBM71-2 caused by the presence of 1 mM LacNAc. (C) A representative of three ITC experiments where 5 mM LacNAc was titrated into 200 µM CBM71-1 in PBS at 25°C. (D) A representative of three ITC experiments where 5 mM lactose was titrated into 200 µM CBM71-1 in PBS at 25°C. (E) A representative of three ITC experiments where 5 mM LacNAc was titrated into 200 µM CBM71-2 in PBS at 25°C. (F) A representative of three ITC experiments where 5 mM lactose was titrated into 200 µM CBM71-2 in PBS at 25°C. For all ITC experiments the solid line represents the best fit from non-linear regression analysis using a one-site binding model.
(DOCX)

Figure S5 **Adherence of R6 and C06_18 to D562 cells can be reduced in the presence of galactose β-1,4 linked or recombinant CBM in a BgaA-dependent manner.** (A) Adherence of *S. pneumoniae* strain R6 and R6Δ*bgaA* to D562 cells in the presence of CBM71-1, CBM71-2 or CBM71-1.2 (250 µM). Asterisks indicate significant differences in adherence in the presence or absence of recombinant CBM. (B) Adherence of *S. pneumoniae* strain CO6_18 and CO6_18Δ*bgaA* to D562 cells in the presence of CBM71-1, CBM71-2 or CBM71-1.2 (250 µM). Asterisks indicate significant differences in adherence in the presence or absence of recombinant CBM. (C) Adherence of *S. pneumoniae* strain R6 and R6Δ*bgaA* to D562 cells in the presence of LacNAc and lactose (0–10 mM). Asterisks indicate significant differences in adherence in the presence or absence of disaccharide. (D) Adherence of *S. pneumoniae* strain CO6_18 and CO6_18Δ*bgaA* to D562 cells in the presence of LacNAc and lactose (0–10 mM). Asterisks indicate significant differences in adherence in the presence or absence of disaccharide. (E) Monosaccharide constituents of LacNAc have no or moderate effect on pneumococcal adherence. Adherence of pneumococci to D562 cells was assessed in presence of 10 mM GlcNAc, galactose (Gal), or LacNAc. GlcNAc has no effect on pneumococcal adherence, while Gal reduces pneumococcal adherence but not to the same extent as LacNAc. Data presented here are mean ± SD of three independent experiments each performed in triplicate. Statistically significant differences were assessed using a two-tailed Student's *t*-test. * p≤0.03, ** p≤0.007 and *** p≤2×10^−4^.
(PPTX)

Figure S6 **BgaA encoded by R6BgaAW1514A,W1864A is appropriately localized and has β-galactosidase activity not significantly different from the parental strain.** (A) R6BgaAW1514A,W1864A is localized to the bacterial cell surface. Immunoblot of cytoplasmic (CP) and cell wall (CW) protein fractions for localization of BgaA expressed by parental strain (R6) and R6BgaAW1514A,W1864A. Mutation of tryptophan residues 1514 and 1864 in strain R6BgaAW1514A,W1864A does not alter the expression and localization of the protein. (B) R6BgaAW1514A,W1864A is not significantly altered in β-galactosidase activity compared to the parental strain. Data are the means ± SD of three independent experiments performed in triplicate. * Indicates a statistically significant difference between R6 and R6Δ*bgaA* using a two-tailed Student's *t*-tests (p≤4.3×10^−5^).
(PPTX)

Figure S7 **Alignment of BgaA from different streptococcal species.** Alignment of the predicted amino acid sequence of BgaA from *S. pneumoniae* (Sp, R6 NP_358159), *Streptococcus oralis* (So, strain Uo5, YP_004325702), *S. gordonii* (Sg, strain CH1, YP_001450765), *Streptococcus parasanguinis* (Sps, strain FW213, YP_006310746) and *Streptococcus mitis* (Sm, strain B6, YP_003446636). Black shading indicates identical amino acid residues and grey shading similar residues. The green underlining indicates amino acids within the GH2 region and the red underlining indicates the CBMs.
(DOCX)

Methods S1 **Supplemental methods.** This text includes additional details of methods used.
(DOCX)

Table S1 **Bacterial strains and plasmids used in the study.**
(DOCX)

Table S2 **Primers used in the study.**
(DOCX)

Table S3 **X-ray data collection and structure statistics.** Values in parentheses are for the highest resolution bin.
(DOCX)
