# Supplementary material for: Unravelling the Multiple Functions of the Architecturally Intricate Streptococcus pneumoniae β-galactosidase, BgaA
Source: PLoS Pathog. 2014 Sep 11;10(9):e1004364. doi: 10.1371/journal.ppat.1004364 (PMC4161441; doi:10.1371/journal.ppat.1004364)
Supplement: Figure S1 — Representative electron density for substrate bound to BgaA and comparison of S. pneumoniae BgaA with E. coli LacZ. (A) LacNAc is represented in blue colored sticks with maximum likelihood/σA weighted 2Fo-Fc electron density map contoured at 0.26 electrons/Å3. (B) Cartoon representation of the structure of the BgaA catalytic region comprising domains I–V and colored sequentially as gray, yellow, purple, blue, and orange is overlapped with E. coli LacZ (tan). (PDF) [file ppat.1004364.s001.pdf]

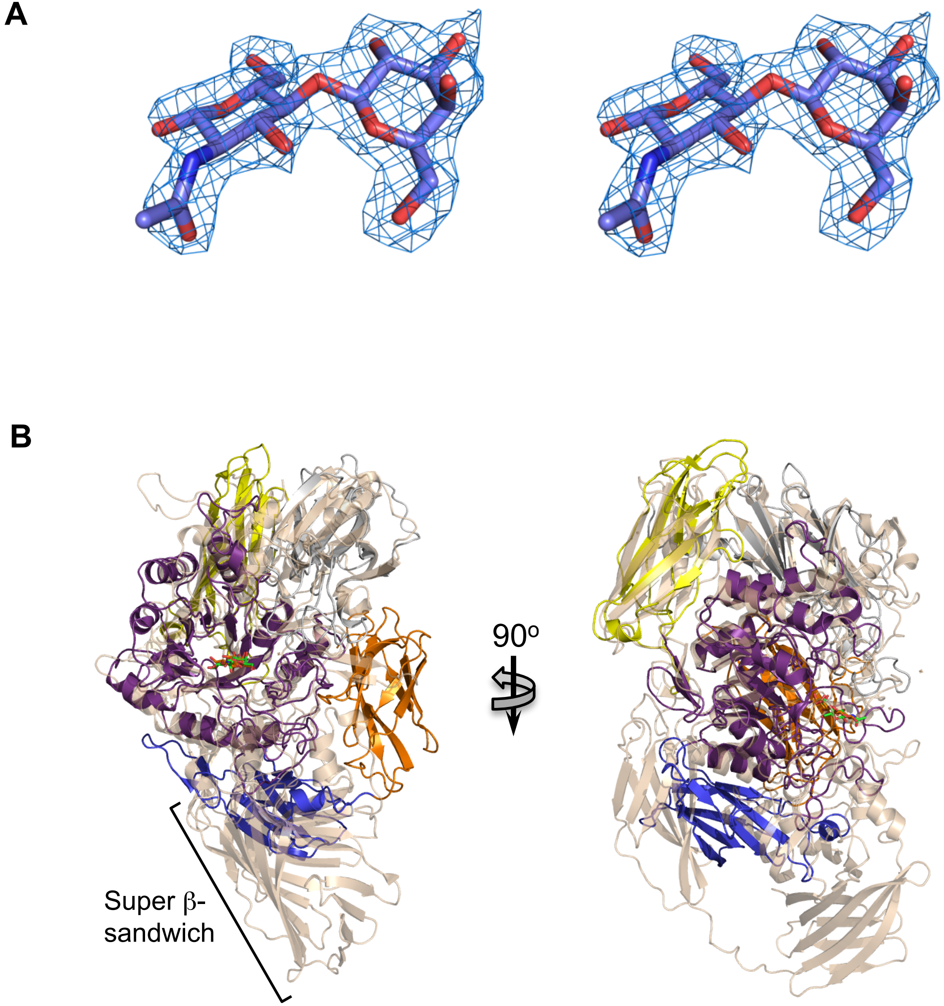
A
B
90°
Super β-
sandwich
