# Supplementary figures and images for: Unravelling the Multiple Functions of the Architecturally Intricate Streptococcus pneumoniae β-galactosidase, BgaA
Source: PLoS Pathog. 2014 Sep 11;10(9):e1004364. doi: 10.1371/journal.ppat.1004364 (PMC4161441; doi:10.1371/journal.ppat.1004364)

**A**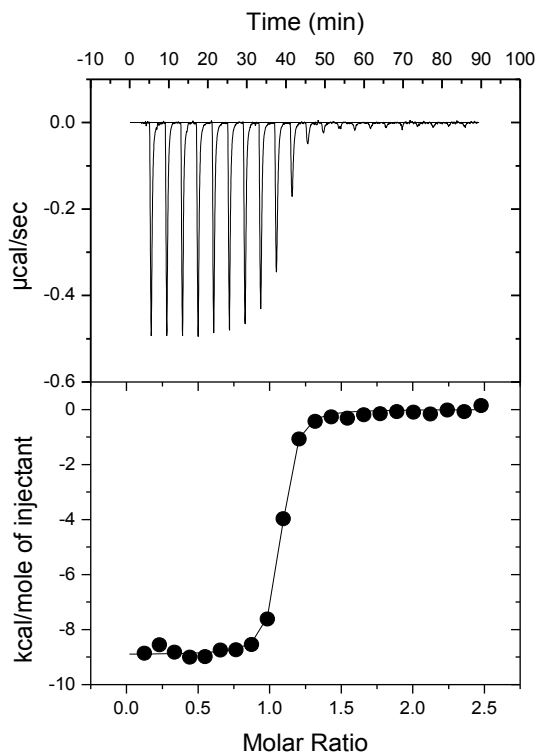**B**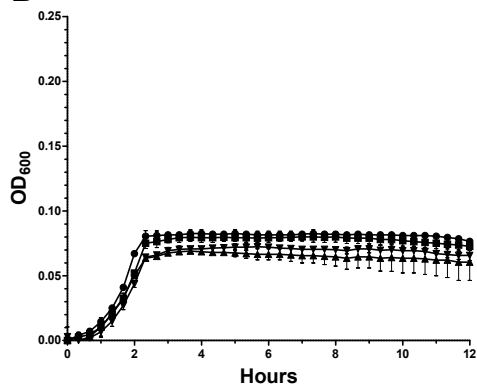**C**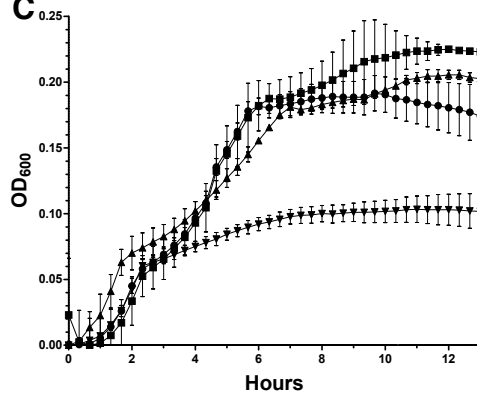**D**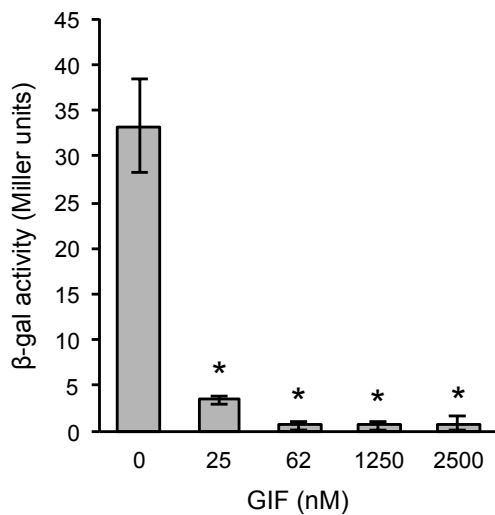**E**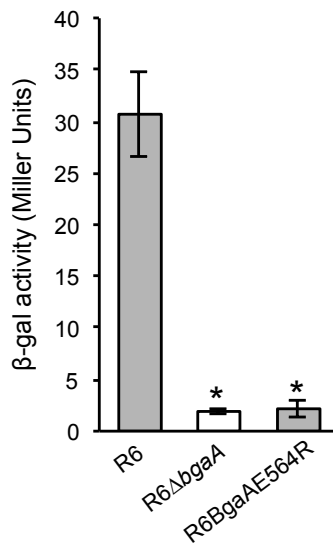

Supplement: Figure S2 — Inhibition of BgaA. (A) Representative ITC titration for GIF titrated into the BgaA catalytic module. The solid line represents the best fit from non-linear regression analysis of using one-site binding model. (B) S. pneumoniae growth controls using a semi-defined medium with no carbon source. Circles represent growth of the TIGR4 strain supplemented with 1 µM purified BgaA catalytic domain, squares growth of the ΔbgaA strain supplemented with 1 µM purified BgaA catalytic domain, triangles the growth of TIGR4 strain, and inverted triangles the growth of the ΔbgaA strain. Error bars represent the standard deviation of triplicate experiments run in parallel. The experiment was performed multiple times with highly similar results. (C) S. pneumoniae growth controls using a semi-defined medium supplemented with bovine asialofetuin. Symbols are as above. (D) Activity of the cell-surface associated BgaA is significantly reduced in the presence of GIF (25–2500 nM). Data presented here are mean ± SD of three independent experiments each performed in triplicate. *Statistically significant reduction in β-galactosidase activity as compared to R6 in the absence of GIF (p≤0.0006). (E) Activity of R6BgaAE564R is significantly reduced as compared to the parental strain. Data presented here are mean ± SD of three independent experiments each performed in triplicate. *Statistically significant reduction in β-galactosidase activity (p≤0.0003) as compared to R6. (PDF) [file ppat.1004364.s002.pdf]

## Slide 1
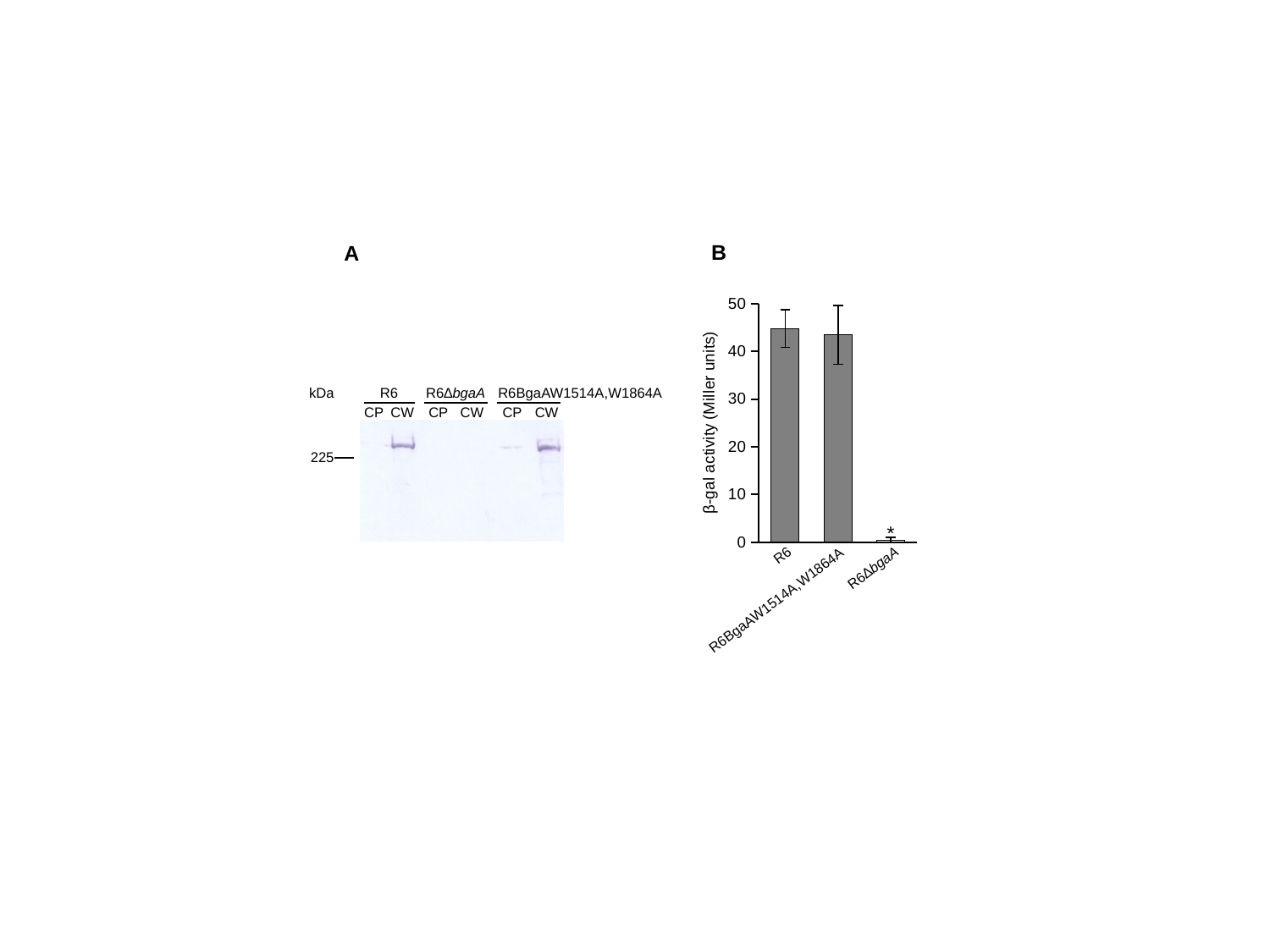

B
### Chart
| Category | |
|---|---|R6
R6∆bgaA
R6BgaAW1514A,W1864A
A
kDa
R6
R6∆bgaA
R6BgaAW1514A,W1864A
CP
CW
CP
CW
CP
CW
225
*

Supplement: Figure S6 — BgaA encoded by R6BgaAW1514A,W1864A is appropriately localized and has β-galactosidase activity not significantly different from the parental strain. (A) R6BgaAW1514A,W1864A is localized to the bacterial cell surface. Immunoblot of cytoplasmic (CP) and cell wall (CW) protein fractions for localization of BgaA expressed by parental strain (R6) and R6BgaAW1514A,W1864A. Mutation of tryptophan residues 1514 and 1864 in strain R6BgaAW1514A,W1864A does not alter the expression and localization of the protein. (B) R6BgaAW1514A,W1864A is not significantly altered in β-galactosidase activity compared to the parental strain. Data are the means ± SD of three independent experiments performed in triplicate. * Indicates a statistically significant difference between R6 and R6ΔbgaA using a two-tailed Student's t-tests (p≤4.3×10^−5^). (PPTX) [file ppat.1004364.s006.pptx]
