# Supplementary material for: Unravelling the Multiple Functions of the Architecturally Intricate Streptococcus pneumoniae β-galactosidase, BgaA
Source: PLoS Pathog. 2014 Sep 11;10(9):e1004364. doi: 10.1371/journal.ppat.1004364 (PMC4161441; doi:10.1371/journal.ppat.1004364)
Supplement: Figure S3 — Determining the role of N and C terminal regions of BgaA in pneumococcal adherence. (A) R6BgaAC has significantly higher adherence to D562 cells as compared to R6ΔbgaA. Adherence of R6BgaAN is not significantly different when compared to R6ΔbgaA. (B) C06_18BgaAC has significantly higher adherence to D562 cells as compared to C06_18ΔbgaA. Adherence of C06_18BgaAN is not significantly different when compared to C06_18ΔbgaA. (C) The N-terminal enzymatic module of BgaA expressed by R6BgaAN is localized to the bacterial cell surface. Immunoblot of cytoplasmic (CP) and cell wall (CW) protein fractions for localization of full length BgaA expressed by parental strain (R6) and the BgaA N-terminal enzymatic module expressed by R6BgaAN. Deletion of BgaA amino acids 991–1984 in strain R6BgaAN does not alter the expression and localization of the protein. (D) R6BgaAN has reduced β-galactosidase activity compared to the parental strain; however, the level of activity is significantly higher than that of R6ΔbgaA. Data are the means ± SD of three independent experiments performed in triplicate. Statistically significant differences were assessed using a two-tailed Student's t-tests. * p≤0.03, ** p≤0.002. (DOCX) [file ppat.1004364.s003.docx]

225

76


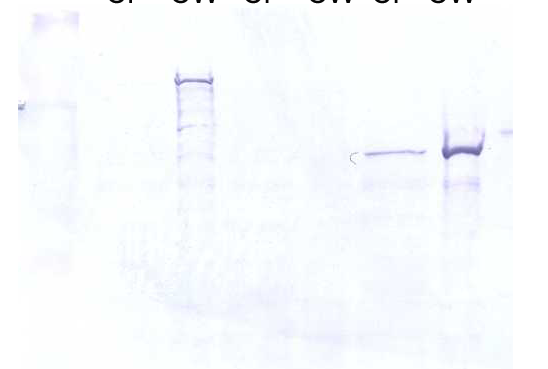


R6

R6∆*bgaA*

R6BgaAN

CP

CP

CP

CW

CW

CW

kDa

**C**


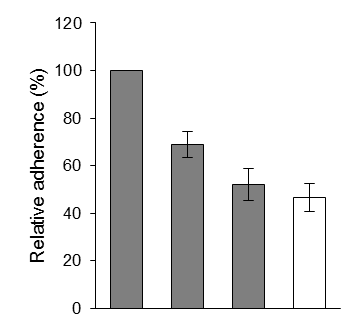


*

C06_18BgaAC

C06_18BgaAN

C06_18∆*bgaA*

C06_18


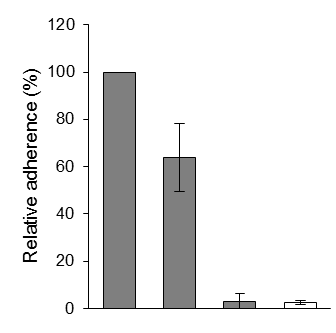


R6BgaAC

R6BgaAN

R6∆*bgaA*

R6

**

**A**

**B**


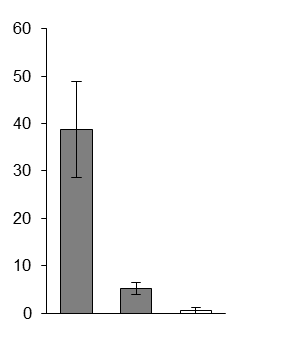


*

*

R6BgaAN

R6∆*bgaA*

R6

β-gal activity (Miller units)

**D**
