# Supplementary material for: Unravelling the Multiple Functions of the Architecturally Intricate Streptococcus pneumoniae β-galactosidase, BgaA
Source: PLoS Pathog. 2014 Sep 11;10(9):e1004364. doi: 10.1371/journal.ppat.1004364 (PMC4161441; doi:10.1371/journal.ppat.1004364)
Supplement: Figure S5 — Adherence of R6 and C06_18 to D562 cells can be reduced in the presence of galactose β-1,4 linked or recombinant CBM in a BgaA-dependent manner. (A) Adherence of S. pneumoniae strain R6 and R6ΔbgaA to D562 cells in the presence of CBM71-1, CBM71-2 or CBM71-1.2 (250 µM). Asterisks indicate significant differences in adherence in the presence or absence of recombinant CBM. (B) Adherence of S. pneumoniae strain CO6_18 and CO6_18ΔbgaA to D562 cells in the presence of CBM71-1, CBM71-2 or CBM71-1.2 (250 µM). Asterisks indicate significant differences in adherence in the presence or absence of recombinant CBM. (C) Adherence of S. pneumoniae strain R6 and R6ΔbgaA to D562 cells in the presence of LacNAc and lactose (0–10 mM). Asterisks indicate significant differences in adherence in the presence or absence of disaccharide. (D) Adherence of S. pneumoniae strain CO6_18 and CO6_18ΔbgaA to D562 cells in the presence of LacNAc and lactose (0–10 mM). Asterisks indicate significant differences in adherence in the presence or absence of disaccharide. (E) Monosaccharide constituents of LacNAc have no or moderate effect on pneumococcal adherence. Adherence of pneumococci to D562 cells was assessed in presence of 10 mM GlcNAc, galactose (Gal), or LacNAc. GlcNAc has no effect on pneumococcal adherence, while Gal reduces pneumococcal adherence but not to the same extent as LacNAc. Data presented here are mean ± SD of three independent experiments each performed in triplicate. Statistically significant differences were assessed using a two-tailed Student's t-test. * p≤0.03, ** p≤0.007 and *** p≤2×10^−4^. (PPTX) [file ppat.1004364.s005.pptx]

## Slide 1
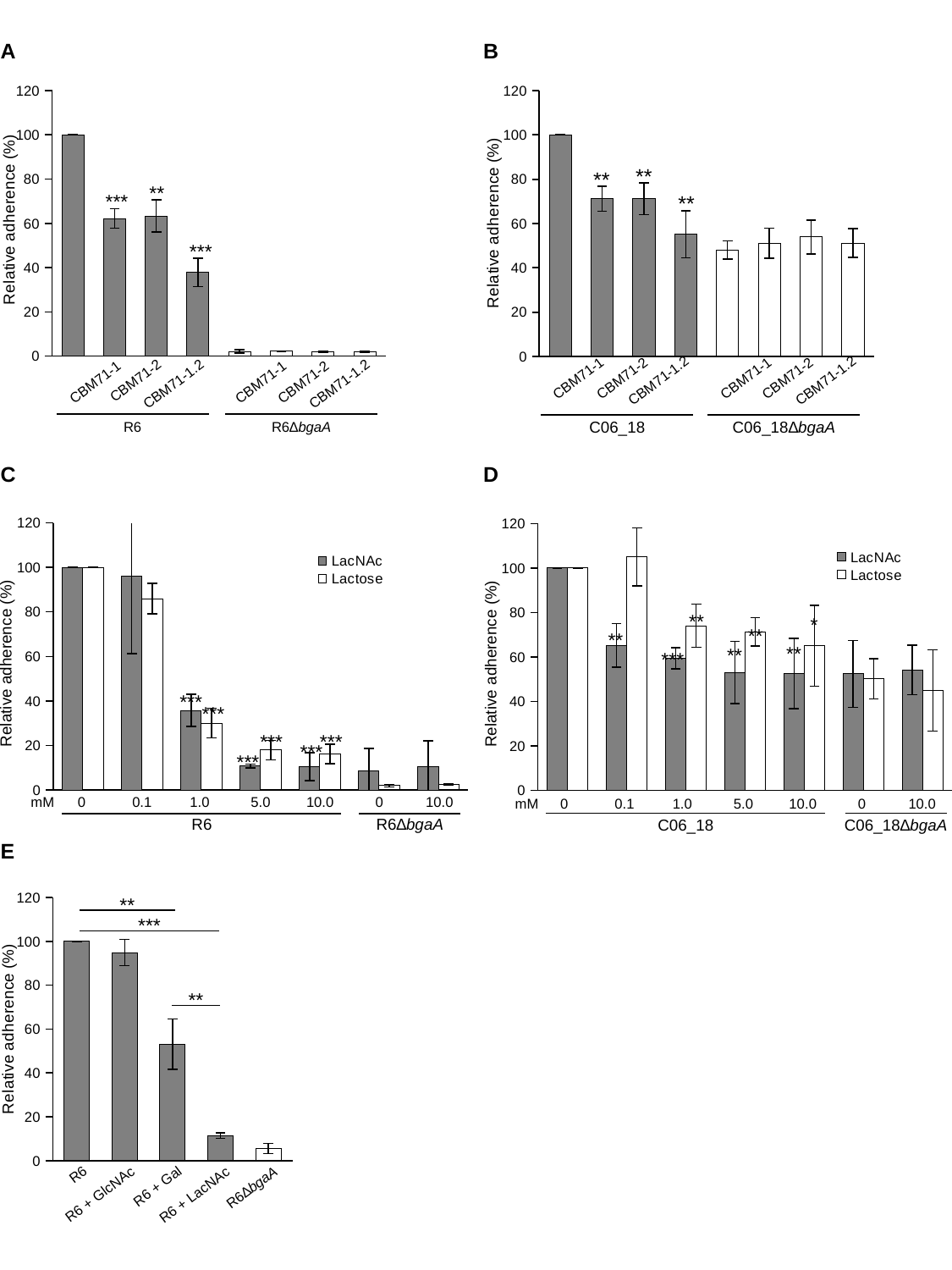

A
B
### Chart
| Category | Avg |
|---|---|
| C06_18 | 100.0 |
| C06_18+CBM1 | 71.17651040980248 |
| C06_18+CBM2 | 71.18861010826426 |
| C06_18+CBM1-2 | 55.13025306309401 |
| C06_18ΔbgaA | 47.98809796448065 |
| C06_18 ΔbgaA+CBM1 | 51.03882781785564 |
| C06_18ΔbgaA+CBM2 | 53.88508204573185 |
| C06_18ΔbgaA+CBM1-2 | 51.166463204379006 |CBM71-1
CBM71-2
CBM71-1
CBM71-2
CBM71-1.2
CBM71-1.2
C06_18
C06_18∆bgaA
### Chart
| Category | |
|---|---|CBM71-2
CBM71-1
CBM71-1
CBM71-2
CBM71-1.2
CBM71-1.2
R6
R6∆bgaA
**
**
**
**
***
***
C
D
### Chart
| Category | LacNAc | Lactose |
|---|---|---|Relative adherence (%)
mM
0
0.1
1.0
5.0
10.0
0
10.0
R6
R6∆bgaA
### Chart
| Category | LacNAc | Lactose |
|---|---|---|Relative adherence (%)
mM
0
0.1
1.0
5.0
10.0
0
10.0
C06_18
C06_18∆bgaA
**
*
**
**
**
**
***
***
***
***
***
***
***
E
### Chart
| Category | |
|---|---|
| R6 | 100.0 |
| R6+10mM GlcNAc | 94.88726586903306 |
| R6+10mM Gal | 53.134255045692406 |
| R6+ 10mM LacNAc | 11.381867903648248 |
| R6∆bgaA | 5.564255089174945 |R6
R6 + Gal
R6∆bgaA
R6 + GlcNAc
R6 + LacNAc
**
***
**
