# Supplementary material for: Unravelling the Multiple Functions of the Architecturally Intricate Streptococcus pneumoniae β-galactosidase, BgaA
Source: PLoS Pathog. 2014 Sep 11;10(9):e1004364. doi: 10.1371/journal.ppat.1004364 (PMC4161441; doi:10.1371/journal.ppat.1004364)
Supplement: Figure S7 — Alignment of BgaA from different streptococcal species. Alignment of the predicted amino acid sequence of BgaA from S. pneumoniae (Sp, R6 NP_358159), Streptococcus oralis (So, strain Uo5, YP_004325702), S. gordonii (Sg, strain CH1, YP_001450765), Streptococcus parasanguinis (Sps, strain FW213, YP_006310746) and Streptococcus mitis (Sm, strain B6, YP_003446636). Black shading indicates identical amino acid residues and grey shading similar residues. The green underlining indicates amino acids within the GH2 region and the red underlining indicates the CBMs. (DOCX) [file ppat.1004364.s007.docx]

Sp 1 ----MGKGHWNRKRVYSIRKFAVGACSVMIGTCAVLLGGNIAGESV--------------
So 1 ----MEKGHWNRKRVYSIRKFAVGACSVMIGTCAVLFGGSVIGESP--------------
Sg 1 ----MEKGYWNRKRVYSIRKFTVGACSVLIGTCAVLFGASLSAGNP--------------
Sps 1 ----MGKSFFEKRPVFSIRKLSVGACSVVIGISALGLSRVHAEEKP--------------
Sm 1 MRILMNKRLFDKRCHYSIRKFAIGVASVMIGASIFGISAVQAEEAASSNTQTEETTVHQA


Sp 43 -----------------------------------VYADETLITHTAEKPKEEKMIVEEK
So 43 -----------------------------------VFADETPIAHTVEQAKEESPAVEEK
Sg 43 -----------------------------------VYAEEVAVNASAESVKEEGMIEEEN
Sps 43 -----------------------------------ALESEPTSIETPSVVTENS------
Sm 61 QPLDKLPDDVAAAIAKADENGGREFVKPKSELAEDKVTKDTETTRPANDGSHELASPKVE


Sp 68 ADKALETKNVVERTEQSEPSSTEAIASEKKEDEAVTPKEEKVSAKPEEKAPRIESQASSQ
So 68 EDQTVAEHKDVASVDQSQAAPIEASKPEKKEDEPVAPKEEKASLKPEETAPKVESQASSQ
Sg 68 SDKVVATSELSEAADASPALSTQETFPEKTEENSPSPKIEKEFSESVKNEAKEISESSAQ
Sps 62 ------------------ETEVPEAGARVSEAPAVITPTRAEEKPAPQGEGQPVSASSER
Sm 121 TPNKVEEGNKAEDKQKSEEANPKPVESAVTAGTEVRDDAKKTSEKDQVKQTTDIKSSSEK


Sp 128 EKPLKEDAKAVTNEEVNQMIENRKVDFNQNWYFKLNANSKEAIKPDADVSTWKKLDLPYD
So 128 EKPVKEDLKAATNEEVNQMIEDRKVNFNQNWHFKLNANPKEAVKSDADVSTWQKLDLPHD
Sg 128 EGSEKDKMKSATEEEVSQMIEDRKVNFNQNWHFKLNANAKEAVKEEADVTSWKKLDLPHD
Sps 104 ATGTEETAAPDQNRVAEDIVQDRERDFNKDWYFKLNAAPG-AEGRQVDVKDWKKLDLPHD
Sm 181 TQALSKESSKADVEKEKQLLSDRKQDFNKDWYFKLNAQGDFSKKD-VDVHDWSKLNLPHD


Sp 188 WSIFNDFDHESPAQNEGGQLNGGEAWYRKTFKLDEKDLKKNVRLTFDGVYMDSQVYVNGQ
So 188 WSIFNDFDHQSPAQNEGGQLNGGEAWYRKTFKLDEKDLKKNVRVTFDGVYMDSQVYVNGQ
Sg 188 WSIHFDFDQDSPAQNEGGQLNGGDAWYRKPLKLDEKDLDKNVRLTFDGVYMDSQVYVNGQ
Sps 163 WSIFFDFDHNSPAQNEGGQLNGGDAWYRKTFRLDDKDLDKKVRLEFGGVYMDSKVYINGQ
Sm 240 WSIYFDFDHKSPARNEGGQLNGGTAWYRKIFTLNEADKNKDVRINFDGVYMDSKVYVNGK


Sp 248 LVGHYPNGYNQFSYDITKYLYKDGRENVIAVHAVNKQPSSRWYSGSGIYRDVTLQVTDKV
So 248 LVGHYPNGYNQFSYDITKYLHKDGRENVIAVHAVNKQPSSRWYSGSGIYRDVTLQMTDKV
Sg 248 LVGHYPNGYNQFSYDITNFLHKDGRENIIAVHVINKQPSSRWYSGSGIYRDVSLLIRDKV
Sps 223 FVGHYPNGYNAFSYDITPYLNADGSENTIAVHVVNQQPSSRWYSGSGIYRDVKLSVTDKV
Sm 300 FVGHYPSGYNHFSYDITEFLNKDGSENSITVQVTNKQPSSRWYSGSGIYRDVTLSYRDKV


Sp 308 HVEKNGTTILTPKLEEQQHGKVETHVTSKIVNTDDKDHELVAEYQIVERGGHAVTGLVRT
So 308 HVEKNGTTILTPKLEQQQHGKVETYVTSKIVNTDDKDHELVAEYQIVERGGQAVTGLVRT
Sg 308 HVQKNGTTILTPQLESQKDGKVDTLVSSKIVNTDDKDHEITAEYQIVRRGGEAVTEVIRT
Sps 283 HLAQYGTTITTPQLEKQQNGAVDTVVKSRIVNQDDQAHSIYAEYEIVDQNGQVVSKKTRS
Sm 360 HVAENGNHITTPKLAEQKNSNVETQVQSKIKNTDKTAANVFVEQQIFTKEGKAVSELVRS


Sp 368 ASRTLKAHESTSLDAILEVERPKLWTVLNDKPALYELITRVYRDGQLVDAKKDLFGYRYY
So 368 ASRTLKAHESTSLDAILEVEQPKLWTVLNDKPALYELITRVYRDGQLVDAKKDLFGYRYY
Sg 368 ASQKLKAHETSTIQTSLQVERPELWTVLNDKPALYELVTRIYRDGQLVDAKKDLFGYRYY
Sps 343 EAQAVLAGQEINLSQTLHVERPKLWNVTDEKAALYTLYTRVYRDSQLVDVQKERFGYRYL
Sm 420 ATKSLAENETAQFNQNIFVNQPTLWTTKSYHPQLYVLKTKVYKEGQLVDVTEDTFGYRYF


Sp 428 HWTPNEGFSLNGERIKFHGVSLHHDHGALGAEENYKAEYRRLKQMKEMGVNSIRTTHNPA
So 428 HWTPNEGFSLNGERIKFHGVSLHHDHGALGAEENYKAEYRRLKQMKEMGVNAIRTTHNPA
Sg 428 NWTPNEGFSLNGQRIKFHGVSLHHDHGALGAEENYKAEYRRLKQMKEMGVNSIRTTHNPA
Sps 403 NWTPEGGFFLNGVATKFHGVSLHHDHGALGAEENYKAEYRRLKQMKDMGVNAIRTTHNPA
Sm 480 NWTAKEGFSLNGERMKFHGVSIHHDNGALGAEENYKATYRKLKLLKDMGVNSIRTTHNPA


Sp 488 SEQTLQIAAELGLLVQEEAFDTWYGGKKPYDYGRFFEKDATHPEARKGEKWSDFDLRTMV
So 488 SPQTLQIAAELGLLVQEEAFDTWYGGKKPYDYGRFFEKDATHPEARKGEKWSDYDLRTMV
Sg 488 SEQTLQIAAELGLLVQEEAFDTWYGGKKPYDYGRFFEKDATHPEAQKGEKWSDYDLRTMV
Sps 463 SEQTLQIAAELGLMVQEEAFDTWYGGKKQYDYGRFFEKDATHPEARKGDTWSDYDLRTMV
Sm 540 SPQLLDAAASLGLLVQEEAFDTWYGGKKTYDYGRFFDQDATHPEAKKGEKWSDFDLRTMV

Sp 548 ERGKNNPAIFMWSIGNEIGEANGDAHSLATVKRLVKVIKDVDKTRYVTMGADKFRFGNGS
So 548 ERDKNNPAVFMWSIGNEIGEANGNAHSLATVKRLVKVIKDVDTTRYVTMGADKFRFGDGS
Sg 548 ERGKNNPAIVMWSIGNEIGEANGDAHSLATVKRLVKVIKSVDKTRYVTMGADKFRFGDGS
Sps 523 ERGKNNPAIVMWSIGNEVGEADGSDKSVATVRRLVKTIKEVDATRYVTMGADKFRFGDGS
Sm 600 ERDKNNPSIVMWSLGNEVEEANGSPHSIETAKRLKAVIKAIDTERYVTMGENKFSRAS-T


Sp 608 GGHEKIADELDAVGFNYSEDNYKALRAKHPKWLIYGSETSSATRTRGSYYRPERELKHSN
So 608 GDHEKIANELDAVGFNYSEDNYKKLRAKHPNWLIYGSETSSATRTRGSYYRPDQELVHSN
Sg 608 GGHEKIADELDAVGFNYSEDNYKALRAKHPNWLIYGSETSSATRTRGSYFHPEQEWVGSN
Sps 583 GGHEKVAAELDAVGFNYSEANYESLRAKHPNWLIYGSETSSATRTRGSYYHPEREWVGSN
Sm 659 GDFLKVAEIMDAVGMNYGERNYDAVRRAHPDWLIYGSETSSATRTRDSYYNPARILGHDN


Sp 668 GPERNYEQSDYGNDRVGWGKTATASWTFDRDNAGYAGQFIWTGTDYIGEPTPWHNQNQTP
So 668 QAYRNYEQSDYGNDRVGWGKTATASWTFDRDNAGYAGQFIWTGTDYIGEPTPWHNQNHTP
Sg 668 QSWRNYEQSDYGNDRVGWGKTATASWTFDRDNAGYAGQFIWTGTDYIGEPTPWHNQNSTP
Sps 643 QEDRHYEQSDYGNDRVGWGRTATASWTFDRDHAGYAGQFIWTGTDYIGEPTPWHNQNDTP
Sm 719 SPNRHYEQSDYGNDRVGWGRTATESWTFDRDHAGYAGQFIWTGIDYIGEPTPWHNQDNTP


Sp 728 VKSSYFGIVDTAGIPKHDFYLYQSQWVSVKKKPMVHLLPHWNWENKELASKVADSEGKIP
So 728 VKSSYFGIVDTAGIPKHDFYLYQSQWVSVKKKPMVHLLPHWNWENRDLASKVEDAQGKIP
Sg 728 VKSSYFGIVDTAGIPKNDYYLYQSQWVSVKKKPMVHLLPHWNWQDQELADNVADAENKIP
Sps 703 VKSSYFGIVDTAGLPKNDFYLYQSQWVSAEKHPMVHLLPHWNWDNPELANRVMDEEGRIP
Sm 779 VKSSYFGIIDTAGLPKNDFYLYRSEWYSAKEKPTVRILPHWNWTDETLRDRKMLVDGKVP


Sp 788 VRAYSNASSVELFLNGKSLGLKTFNKKQTSDGRTYQEGANANELYLEWKVAYQPGTLEAI
So 788 VRAYSNAASVELFLNGQSLGVKKFNKKQTSDGRTYQEGANAKELYLEWKVAYQPGTLEAV
Sg 788 VRAYSNAASVELFLNNQSLGLKKFNKKETSDGRSYQEGANPQELYLEWKVAYQPGTLEAV
Sps 763 VRAFSNAHSVELIVNGESQGVKTFTKKTTADGRTYQEGANPDELYLEWLVPYVPGKVEAI
Sm 839 VRTFSNAANVELFLNGESLGKKEFTKKRTEDGRPYHEGAKPSELYLEWLVKYQPGTLTAI


Sp 848 ARDESGKEIARDKITTAGKPAAVRLIKEDHAIAADGKDLTYIYYEIVDSQGNVVPTANNL
So 848 ARDEAGKEIARDKITTAGQPAGVRLIKEEHAIAADGKDLTYIYYEIVDSEGNVVPTANNL
Sg 848 ARDDQGKEIARDKIVTAGQPAGVRLVKEEHAIAADGKDLTYIYYEIVDRDGNVVPTANNL
Sps 823 ARNEAGEVIAKDQVETAGKPAGVRLLKEEYAIAADGKDLTYITYEIVDEEGRVVPTANNL
Sm 899 ARDENGNEIARDSVTTAGEPARVRLTKEEHVITADGKDLSYIHYEIVDGEGNVVPTANNL


Sp 908 VRFQLHGQGQLVGVDNGEQASRERYKAQADGSWIRKAFNGKGVAIVKSTEQAGKFTLTAH
So 908 VRFQLHGQGQLVGVDNGEQASRERYKAQPDGSWIRRAFNGKGVAIVKSTEQAGKFTLTAH
Sg 908 VRFQLHGQGQLVGVDNGEQASRERYKEQADGSWIRKAFNGKGVAIVKSTDQAGKFTLTAH
Sps 883 VHFHLHGQGQIVGVDNGEQASRERYKAQEDGSWQRKSFNGKGVVIVKSTEQAGAFTLYAD
Sm 959 VHFNLHGQGQIVGVDNGEQASRERYKAQADGTWQRRAFNGKGVVIVKSTEKEGKFTLYAD


Sp 968 SDLLKSNQVTVFTGKKEGQEKTVLGTEVPKVQTIIGEAPEMPTTVPFVYSDGSRAERPVT
So 968 SDLLKSSQVTVFTGKKEDQEKTVLGTEVPKVRTVIEKEPKMPKTVGFIYSDGSREKRPVT
Sg 968 SDLLKSDQVTIFTGKNDQKDKTVLGTEVPKVRTVLGQAPSLPATVPFVYSDGSRTEQPVT
Sps 943 SDRLQSDQVSLFTGKKDQAERTVLGVEPVRQSAYLGEEPTLPSKVNVVYSDGKAQEEAVE
Sm 1019 SAGLTSDSATVATVSGKKENRHFIAYAPVKATTDVSTTPELPRTVTAIYSDGSVEEKDVT


Sp 1028 WSLVDVSKPGIVTVKGMADGR----EVEARVEVIALKSELPVVKRIAPNTNLNSVDKSVS
So 1028 WSSVDVSQAGVVTVKGMADGR----EVEARVEILAIANELPTVKRVAPGTDLSAVDKYVS
Sg 1028 WSQADVSHSGIVTVKGESEGR----QVEARVEVLAVERELPVIKRVAPGTDLSAVDKSVA
Sps 1003 WDAADYSRAGQVRVTGHVQGR----TVEALVDVIGVEQVLPVIKQIPQGADLATVDKAVQ
Sm 1079 WKVPSDLLTSAGEKKVFGNVEGLEAKAEALVKVVALDKWLPKVATVPVGTTAADLDKTVT


Sp 1084 YVLTDGSVQEYEVDKWEIAEEDKAKLAIPGSRIQATGYLE-GQPIHATLVVEEGNPAAPV
So 1084 IAVTDGSVQEYEVDKWEIAEADKAKLSVAGSRIQMTGQLG-GETIHATLVVEEGEAAAPV
Sg 1084 LALTDGSLEHYDVDKWEIAPEDKEKLSVPGSRIQMTGQLA-GTKVSATLLVTDREAEAPV
Sps 1059 LVFTDGTTAHYEVDQWTLEAGQEDQLSTPGARLNATGLLGNGETVQATLVVAGGDVAKAK
Sm 1139 AVLSDGSLIDTDVVSWTLKDP--AALNKEGGRTEATGKLVDDDREVTATFIASSKETTSS

Sp 1143 VPTVTVGGEAVTGLTSRQPMQYRTLSYGAQLPEVTASAENADVTVLQASAANGMRASIFI
So 1143 APTVTVGGESVAGLTSQNPMQYRTLAYGASLPEVVASAENAAVTVVQASAANGMRASIFV
Sg 1143 NPVVTLDGDSVTGLTSQKPVHYHSLAYGSQLPLVAATAENADVTVVQASVANGLRASIYV
Sps 1119 KPTVQLDGVALPKFGSGNHTIFRSLAYGQEPGQVTASAENAQVTVLQANRENGLKAQIYV
Sm 1197 ITGLTVGDKTLENFESGKTYYRVSLPYTATIPSVGAQTTGYQVTVQQASADNGYQASVFL


Sp 1203 QPKDGGPLQTYAIQFLEEAPKIAHLSLQVEKADSLKEDQTVKLSVRAHYQDGTQAVLPAD
So 1203 QPNDGGPLQMYAIQFLEEAPKIDHLSLQVEQADGLKEDQTVKLSVLAHYQDGTQAVLPAD
Sg 1203 QPKDGGTLQTYAVQFLEEAPKIERLSLQVEQANHLKEDQTVQLTVLAHYQDGTQAVLQAD
Sps 1179 TAKDTGAVQTYVVQFQEESPQIERLELRLPEGQELKEDQTVPLEIIAHYQDGSLASLKAD
Sm 1257 SDQKGDLVQTYLIQFVKEAPALTRLEVSVEGKEKATEDQVLPYHVIGHYEDGSQTEFAAS


Sp 1263 KVTFSTSG--EGEVAIRKGMLELHKPGAVTLNAEYEGAKG-----QVELTIQANTEKKIA
So 1263 KVNFSTSG--EGEVAVRKGMLELHKPGTVTLKAEYEGARG-----QIDLTIQANTEKKIA
Sg 1263 KVAFSTTG--EGELAVRKGMLELHKPGIVTLKAEYEGAEG-----QVNLTIQANTENKTV
Sps 1239 QIDVKTRAGSQGKAVATKKGLELREAGLVHLEANFQGQTG-----EVSFTITPNPEEKTV
Sm 1317 DVHLEAKSADGGHLEVNGQNLLLYTKGRVTLTPRIDNQTEKTKSVATELVIKENKVEKKI


Sp 1316 QSIRPVNVVTDLHQEPSLPATVTVEYDKGFPKTHKVTWQAIPKEKLDSYQIFEVLGKVEG
So 1316 QSIRPVNVVTDLHQEPSLPATVTVEYDKGFPKAHKVTWQAIPKEKLDHYQTFEVLGKVEG
Sg 1316 QSVRPVSVVTDLHQEPNLPTTVTVEYDKGFPKVHKVTWQAMAKEELDRYHTFEVVGKVEG
Sps 1294 VKVRPVRISTDRNMLPALPETVLVEYDKGFPKEKRVTWDAVTADQVKDYHSFTVTGHVEG
Sm 1377 VKLHPVSISTDINQQPNLPSQVGAEFDKGLPRKVAVTWDKVAEKELGHYHSFTLKGHVEG


Sp 1376 IDLEARAKVSVEGIVSVEEVSVTTPIAEAPQLPESVRTYDSNGHVSSAKVAWDAIRPEQY
So 1376 IDLEARAKVSVEGIVSVEEVSVTTPIAEAPQLPESVRTYDSNGHVSSAKVTWDKIRPEQY
Sg 1376 IDQKAIAKVSVEGIIAVEEVSVTTPIAEAPHLPESVRTYHSNGQVSSAKVTWEPIAPSQY
Sps 1354 VEKEAQAQVTVEGIIAVEEVSTTTPVGEKPALPESVRTYHSNGKTYTAKVAWDAVDPQLL
Sm 1437 TDIEAQATVTVEGLQVAEEISLTLPKGETVQLPASVRAYHSNGTTIYKDVVWDQVPANFS


Sp 1436 AKEGVFTVNGRLEGTQLTTKLHVRVSAQTEQGANISDQWTGSELPLAFASDSNPSDPVSN
So 1436 AKEGVFTVNGRLEGTQLTTKLHVRVSAQTEKGANISDQWTGSELPLAFASDSNPTDPVSN
Sg 1436 QKEGVFTVSGQVEGSSLPTKLHVRVSAQTENGANISDQWTGSELPLAFASDSNPSDSVSN
Sps 1414 AKEGEVILSGRVEGTDLSTRLHIRVSANTVKGANVAEQWTGSVLPLAFASDSNDADPVAK
Sm 1497 QTEGVYEINGHLVGSNLTTKAHVRVSSQVVAGNNISKQWTGSQLPAAIVSNTGGDDSANA


Sp 1496 VNDKLISYNNQPAN-RWTNWNRSNPEASVGVLFGDSGILSKRSVDNLSVGFHEDHGVGAP
So 1496 VNDKLISFNDRPAN-RWTNWNRTNPEASVGVLFGDSGILSKRSVDNLSVGFHEDHGVGVP
Sg 1496 VNDKVISYTDQPAN-RWTNWNR-REEDSVGVLFGDSGILTKRSVDNLNVAFHEDHGVGAP
Sps 1474 VNDKVISFTDAPAN-RWTNWGRDNAEDSVGILFGDSGILTKRAVDNLHVGFHEDHGVGAP
Sm 1557 LNDLTVSRTPTDAKNRWTTWRTNTDNDWASILFGNSGDLTKRFVDNLSVDFYTDGAIGLP


Sp 1555 KSYVIEYYVGKTVPTAPKNPSFVGNE-DHVFNDSANWKPVTNLKAPAQLKAGEMNHFSFD
So 1555 KSYVIEYYVGKTVPTAPKNPSFVGEE-NHAFNDPANWKEVSNLKAPAQLKAGEMNHFSFD
Sg 1554 KSYVIEYYVGQATPTAPKNPSFVESE-EHVFNDDSNWKPVTNLKAPDQLKAGEMNHFNFD
Sps 1533 SEYVIEYYTGEQIPTVPSNPNRVKDETDHPFNNPANWKEVSNLTVEEPVAAGKMNHFSFD
Sm 1617 KEYVIEYYVGQEVPDLPNDVNNAQRDSNHPFNNAANWKEVEHLKAPGQLSAGKTNHFTFD


Sp 1614 KVETYAIRIRMVKADNKRGTSITEVQIFAKQVAAAKQGQTRIQVDGKDLANFNPDLTDYY
So 1614 KVETYAVRIRMVRLDSKKGTSITEVQIFAKQVAAAKQGQTRIQVDGKDLANFNPDLTDYY
Sg 1613 KVDTYAVRIRMVRADDKLGTSITEVQIFSKQVAPAKQAQTRIQVAGQDLPNFNPNLTDYY
Sps 1593 KVDTYAVRIRMKTPEGKRGSSISEIQIFANKVAAEEKSQLTIRVNGDVLPGVNPSVTDYY
Sm 1677 KVETYAVRMRMKKADGTAGIGLTELTVLGNKVVSSTSSEISIKVDGKDLEHFNPSKTDYY


Sp 1674 LESVDGKVPAVTANVSNNGLATVVPSVREGEPVRVIAKAENGDILGEYRLHFTKD-KNLL
So 1674 LESVDGKVPAVTASVSNNGLATVVPSVREGEPVRVIAKAENGDILGEYRLHFTSN-KDLL
Sg 1673 LEAKDGKADEVTASVSNNGLATVVPSVREGDPVRVIVKAENGDILGEYRIHFTKD-KDLL
Sps 1653 IDARDRAYPQVEATASHHGLATVVPSVHEGEPIRVIHKAEDGTILQEYRLHLTSDTEKLK
Sm 1737 IPQSSK---EITATASNNGLVTVVPATSEKGATRLILKAEDGTVLKEYRIFRDDE---KE

Sp 1733 SHKPVAAVKQARLLQVGQALELPTKVPVYFTGKDGYETKDLTVEWEEVPAENLTKAGQFT
So 1733 SRKPVATVKQARLLQVGQPLELPTKVPVYFTGKDGYETKDLAVEWEEVPAENLTKAGQFT
Sg 1732 ARKPIATVKQARLLQLGQNLELPSKVPVYFTGEKDYEVKDLAVEWEPVPAENLSKAGQFT
Sps 1713 QAAPVAVEAGSRFVKVGQDLVLPSTVGVYFQGDTGYERKELTVDWKPLPADALSHEGSFT
Sm 1791 STQPAAAENSAKTLNVGDQLQLPAEVTVYYPSQAGWVKANLAVQWDAIPEHATEQEGSFE


Sp 1793 VRGRVLGSNLVAEVTVRVTDKLGETLSDNPNYDENSNQAFASATNDIDKNSHDRVDYLND
So 1793 VRGRVLGSDLVAEFTVRVTDKLGESLSDNPNYNENGNRAFASATNDIDKNSKDRIDYIND
Sg 1792 VHGRLVGSNLAVELRVRVTDKLGESVSDNPYYDENSNQAFASETNDADPDSHDRVDYIND
Sps 1773 LEGKVLGYDLTAQLTVRVSEKTGENLSVNRDYNAEGTRAFASETNDLDPNQIDYIDYIND
Sm 1851 VTGHVIGTNLTTKIQVTVVAKGNQVLSENPSNNEMDSKAFASATNDTQAASHDRIFYIND


Sp 1853 GDHSENRRWTNWSPTPSSNPEVSAGVIFRENGKIVERTVAQAKLHFFADSGTDAPSKLVL
So 1853 GNQDESRRWTNWSPTPSSNPEVSAGVIFRENGKIVERTVAQAKLHFFADSGTDAPSKLVL
Sg 1852 GDLDEGRRWTNWSATPSANPDVSAGVIFKENGKIVERTVNQAKLHFFADSGTDAPSKLVL
Sps 1833 GGYNEYYRWTNWKREPD-QTEVFAGLIFKKNGQVTERLVNKVAVDFFADQETGLPTKTVL
Sm 1911 GKYNEDGRWTNWSRTPK-NQETSVGLVFKKDGKIASQSIGKVAIQFFKDSGTDAPEKMVL


Sp 1913 ERYVGPGFEVPTYYSNYQAYESGHPFNNPENWEAVPYRADKDIAAGDEINVTFKAVKAKV
So 1913 ERYVGPEFEVPTYYSNYQAYDAAHPFNNPENWETVSYRADKDIAAGDEINVTFKAVKAKA
Sg 1912 ERYVGPDFDVPIYYSNYRAYESNHLFNNPDNWESVPYNMKQDIQAGTELTATFNAVKSKA
Sps 1892 ERYIGPDFEVPDDYGNLKNLP-DHPFNQASNWEEIPYSLDYDFEAGYISNLSFNETRTKA
Sm 1970 ERYIGPAFTEPSTIS-RYEENADHPFNKAENWASIPYKASGELVAGKPIEFNFEPVQTTA


Sp 1973 MRWRMERKADKSGVAMIEMTFLAP-SELPQESTQSKILVDGKELADFAENRQDYQITYKG
So 1973 MRWRMERKADKSGVAMIEMTFLAP-SELPQESTQSKILVDGNELPDFAEDRQDYQITYKG
Sg 1972 MRWRMDRKANKNGVALIEMTFLAP-SELPKESTKSKILLNGKELPDFSAERLEYQVSYSG
Sps 1951 IRLRMVRDENLKGIGIIELSAYAP-TEEAQATTDVTIQVNGKDLEGFKPDVTDYHLEYEG
Sm 2029 IRARMTRKATTNGLAVVEFTAYSAGKGAEVETPSATISIDGKALENFDPNVTDYTLTAIS


Sp 2032 QRPKVSVEENNQVASTVVDSGEDSLPVLVRLVSESGKQVKEYRIQLTKE---------KP
So 2032 QRPKVSVEESNQVASTVVDSGDDSLPVLVRLVSESGKQVKEYRIQLTKENPV----SEKT
Sg 2031 QRPKVTVEENDQVASTVVDSGDNRLPVLVRLLSESGKQVKEYRIQLTEEKTT----AGKV
Sps 2010 DRPIVSAQGKNGTAVTVVDAKSANAPVLVKVVSEDGKLEKVYQLSLSAKAPTGSAIPEEG
Sm 2089 SQPKVTATTSGHGVVTVVNPGNTNLPTLVRLVSKDGNLVKEYRLHFKTAFQTTP---TEG


Sp 2083 VSAVQEDLPKLEFVEKDLAYKTVEKKDSTLYLGETRVEQEGKVGKERIFTVINPDGSKEE
So 2088 VAAVQEDLPKLEFVEKDLAYKTVEKKDSTLYLGETRVEQEGKVGKERIFTAVNPSGSKEE
Sg 2087 EATVQEELPRLEVEEKELSYQTIEKEDANLYLGESRVEEEGQNGKERIFTEVSPDGQRVE
Sps 2070 VKNLVHTKPELVIEPEEMDFERLERPNADLPKGEKRVVQEGQKGRKLRLVEVSQENGVES
Sm 2146 VKNLIAETPSLEIEKTPLPFKEVIRENPELAQGQRRVVSEGQAGEKVDYIQVS--GATRT


Sp 2143 KLREVVEVPTDRIVLVGTKP----------------------------------------
So 2148 KLREVVEAPTDRIVLVGTKPGTSLPEDEVKNLVLNRPELVIEEETIDFKVQEQKNDQLPA
Sg 2147 KLREVVQDPINRVLLVGIKKGTALPEDDIKDLVLYRPELLIEEEEVDFQVQERKNDMLPV
Sps 2130 RKELDAFVELDPVAEITEVGTKEVLPDTPQSEPQPQPEPQPLPNPETSHVSEGP------
Sm 2204 LVHTEQRKAQDRIIEVGVKPSISNSKGEEPAPVNEVPEFKGGVNFVEASVNEVPEFKGGA


Sp 2163 ----------------------------------------------------VAQEAKKP
So 2208 GQTRVLQEGQKGIRVHLIEVENGKRTEKESYDKVVAQDRIVEVGTAGETTKPVPQESTKP
Sg 2207 GQTRIIQEGRKGIRVNLVELENGKRTLKESYNKLETQDQILEVGTALSEEKPKSQDTYKA
Sps 2184 --------------------------------------VATATPQASLEVKAVRQEGTDA
Sm 2264 NFVEGAVNDISEYKGQQSTVGNQVAPVDEKPDFKDGVNPGVPQDSKLPEDKAAPETVGNQ


Sp 2171 Q--------------------------VSEKADTKPIDSSEADQTNKAQLPNTGSAASQA
So 2268 Q--------------------------VSEKTDTKEIASSAAGQVQKEQLPNTGSAEGQA
Sg 2267 HSQTSEQDKYQSLEEKVQGTKAMKEEHTEAKSEAVQLQAEKVKSTENSHLPNTGSQSDQA
Sps 2206 AG--------------------------EEVEKTAQTPAVSAAPVSEGRLPNTGTEESVA
Sm 2324 EVPVR---------------AKTEHPVQNIANNQTAERKASAVKGDQNSLPETGERESDT

Sp 2205 AVAAGLALLGLSAGLVVTKGKKED---
So 2302 AVAAGLALLGLSAGLVATKGKKED---
Sg 2327 AITTGLALLGLGASLVAGKRRKED---
Sps 2240 SLVAGILAAGLASAVLDDQKKRANKAK
Sm 2369 AIFLAGVSLALSAALLATKRKED----
