# Supplementary material for: Unravelling the Multiple Functions of the Architecturally Intricate Streptococcus pneumoniae β-galactosidase, BgaA
Source: PLoS Pathog. 2014 Sep 11;10(9):e1004364. doi: 10.1371/journal.ppat.1004364 (PMC4161441; doi:10.1371/journal.ppat.1004364)
Supplement: Methods S1 — Supplemental methods. This text includes additional details of methods used. (DOCX) [file ppat.1004364.s008.docx]

**SUPPORTING EXPERIMENTAL PROCEEDURES**

**Materials.** GIF was synthesized as described previously [[1](#_ENREF_1)]. LacNAc and GNJ was purchased from V-Labs and Toronto Research Chemicals, respectively. β-lactose was purchased from Carbosynth (UK). Unless otherwise specified all reagents and chemicals were purchased from Sigma.

**Bacterial strains, plasmids and culture media.** Bacterial strains and plasmids used in this study are described in [Table S1](http://mic.sgmjournals.org/content/157/8/2369.long#T1). *S. pneumoniae* and *S.* *gordonii* strains were routinely grown in Todd–Hewitt broth supplemented with 0.2% yeast extract (both from Becton, Dickinson and Co.) (THY) at 37°C without shaking. Tryptic soy (TS) (Becton, Dickinson, and Co., Sparks MD) plates with 1.5% agar supplemented with 5000 U catalase (Worthington Biochemical, Corporation, Lakewood, NJ) or TS plates supplemented with 5% sheep blood (Becton, Dickinson and Co.) were used for growth on solid surface. Plates were incubated at 37°C in 5% CO_2_. When appropriate, TS plates were supplemented with streptomycin (200 µg ml^−1^), kanamycin (500 µg ml^−1^), chloramphenicol (5 µg ml^−1^), and erythromycin (1 µg ml^−1^).

*Escherichia coli* cells were grown and maintained in Luria–Bertani (LB) medium. For growth on solid surface LB plates with 1.5% agar were used. When required, LB was supplemented with ampicillin (100 µg ml^−1^), and kanamycin (50 µg ml^−1^).

**Cloning.** Gene fragment encoding the BgaA catalytic module with its four surrounding modules (referred as GH2, amino acids 110-985) was amplified by PCR from *S. pneumoniae* TIGR4 genomic DNA (American Type Culture Collection BAA-334D) using specific primers (Table S2) to introduce a 5' *Nhe*I and 3' *Not*I restriction sites. PCR product was digested and ligated to *Nhe*I and *Not*I digested pET28a. Site-directed mutation was introduced to create a catalytically inactive mutant protein using the “megaprimer” PCR method [[2](#_ENREF_2)]. Gene fragments encoding BgaA CBMs (referred to as CBM71-1, amino acids 1463-1645, CBM71-2, amino acids 1813-1998 and CBM71-1.2, amino acids 1463-1998) were amplified using specific primers (Table S2; primers CBM71-1 F and R, primers CBM71-2 F and R, and primers CBM71-1 F and CBM71-2 R) and cloned in pET28a using an InFusion EcoDry cloning kit. The resulting gene fusions encoded an N-terminal six-histidine tag fused to the protein by an intervening thrombin protease cleavage site. Bidirectional DNA sequencing was used to verify the fidelity of each construct.

**Protein expression and purification.** All recombinant expression vectors were transformed into *E. coli* BL21 Star (DE3) cells (Invitrogen) and proteins were produced using LB medium supplemented with kanamycin (50 µg ml^-1^) as previously described (Pluvinage et al., 2011). Briefly, bacterial cells transformed with the pET28a expression vector were grown at 37°C to an optical density of ~ 0.9 at 600 nm. Protein production was then induced by the addition of 0.5 mM isopropyl β-D-1-thiogalactopyranoside (IPTG); following by overnight incubation at 16°C with shaking. Seleno-methionine-labeled CBM71-1 was produced as above using *E. coli* B834 (DE3) as the expression strain (Novagen). The defined media containing seleno-methionine was prepared according to the instructions of the manufacturer (Athena Enzyme). Cells were harvested by centrifugation and disrupted by chemical lysis. Proteins were purified from the cleared cell-lysate by Ni^2+^-immobilized metal affinity chromatography followed by size exclusion chromatography using a Sephacryl S-200 column (GE Healthcare) and cation exchange chromatography (Bio-Rad). Purified protein was concentrated using a stirred-cell ultrafiltration device with a 10K molecular weight cut-off membrane (Millipore).

Protein concentration was determined by measuring the absorbance at 280 nm and using calculated molar extinction coefficient of 174070 cm^-1^ M^-1^ for GH2 and GH2-E645Q, 29540 cm^-1^ M^-1^ for CBM71-1, 33920 cm^-1^ M^-1^ for CBM71-2, and 76320 cm^-1^ M^-1^ for CBM71-1.2, (Gasteiger, 2003).

**Crystallography procedures.** All crystallization experiments were performed the using sitting-drop vapor diffusion for screening and hanging drop vapor diffusion for optimization, all at 18°C. Native crystals of GH2 (25 mg ml^-1^) were obtained in the optimized crystallization conditions comprise 0.1 M Tris pH 8.8, 2.05 M LiSO_4_. To obtain complex structure of GH2 with an inhibitor, native crystals were soaked in the crystallization solution in presence of an excess of GIF or GNJ. Then the crystallization solution was supplemented with 30% (v/v) ethylene glycol as cryoprotectant before flash freezing with liquid nitrogen. Crystals of GH2-E645Q (25 mg ml^-1^) also grew in presence of 0.1 M Tris pH 8.8 and 2.05 M LiSO_4_ and then were soaked in the crystallization solution containing an excess of LacNAc. Prior to flash cooling, the crystals were then cryoprotected by addition of 15% (v/v) 2-methyl-2,4-pentanediol.

Crystals of selenomethionine labeled CBM71-1 (20 mg ml^-1^) were grown in 5% (w/v) polyethylene glycol (PEG) 400, 20% (w/v) PEG 8000, and 0.1 M HEPES pH 7.5. Crystals of unlabeled CBM71-1 in complex with LacNAc were produced by cocrystallization of CBM71-1 (25 mg ml^-1^) with 4 mM LacNAc in 5% (v/v) DMSO, 20% (w/v) PEG 8000, and 0.1 M HEPES pH 7.5. CBM71-2 (25 mg ml^-1^) was crystallized in 0.15 M KBr, 25% (w/v) PEG 2000 monomethyl ether, and 0.25% (w/v) dextran. In all cases, the crystals were cryoprotected prior to flash cooling in the crystallization solution supplemented with 20% (v/v) ethylene glycol.

Diffraction data were collected either on an a home source comprising a Rigaku R-AXIS IV++ area detector coupled to a MM-002 X-ray generator with Osmic "blue" optics and Oxford Cryostream 700, Beamline 9-2 of the Stanford Linear Accelerator Center [SLAC, Stanford Synchrotron Radiation Lightsource (SSRL), California], 08ID-1 (CMCF-ID), or 08BM-1 (CMCF-BM) at the Canadian Light Source (CLS, Saskatoon, Saskatchewan) as indicated in Table S3. All diffraction data was processed using MOSFLM and SCALA (Powell, 1999; Winn et al., 2011). All data collection and processing statistics are shown in Table S3.

The X-ray crystal structure of the BgaA catalytic region was solved by molecular replacement using the coordinates of hypothetical protein BVU_0391 from *Bacteriodes vulgatus* (PDB ID 3GM8) as a search template. Using PHASER [[3](#_ENREF_3)] and the native data a single protein molecule was found in the asymmetric unit (AU). This model was correct by a combination of automated model building with BUCANEER [[4](#_ENREF_4)], manual building with COOT [[5](#_ENREF_5)], and refinement with REFMAC [[6](#_ENREF_6)]. This model was used as a starting point to determine the structures of the BgaA catalytic region in complex with inhibitors (unmutated enzymes) and LacNAc (E645Q mutant).

The structure of CBM71-1 was determined a by single-anomalous dispersion (SAD) experiment optimized for selenium (see Table S3 for the wavelength at which SAD data was collected). The heavy atom substructure was determined using the program ShelXC/D, while phasing was performed using ShelxE (Sheldrick, 2008). CBM71-1 crystallized with two molecules in the AU; two of the potential three selenium sites per monomer were found and used for phasing. Density modification with the program DM [[7](#_ENREF_7)] was used to improve the phases prior to model building. ARP/wARP [[8](#_ENREF_8)] was able to build an almost complete model, which was completed by manual model building with COOT and refinement with REFMAC. The completed model of CBM71-1 was used as a starting point to determine the structure of this CBM in complex with LacNAc.

The structure of CBM71-2 was solved by molecular replacement using the coordinates of CBM71-2 as a search model and PHASER to find the two protein molecules in the AU. Automated model building was carried out with ARP/wARP followed by manual completion with COOT and refinement with REFMAC.

For all structures, water molecules were added using FINDWATERS in COOT and manually checked after refinement. In all data sets, refinement procedures were monitored by flagging 5% of all observation as ‘free’ [[9](#_ENREF_9)]. Model validation was performed with SFCHECK [[10](#_ENREF_10)], PROCHECK [[11](#_ENREF_11)], MOLPROBITY [[12](#_ENREF_12),[13](#_ENREF_13)]. All model statistics are shown in Table S3.

**Enzyme inhibition.** All steady state kinetic studies were performed in triplicate at 37°C in a Cary/Varian 300 Bio UV-Visible Spectrophotometer as previously described (Pluvinage et al., 2011). Standard reaction mixtures for the determination of kinetic constants were done at 37^o^C in 100 mM potassium phosphate buffer, pH 6.5, containing 0.1% bovine serum albumin in 1 ml volumes. Reaction used 10 nM of enzyme with a range of 2-nitrophenyl β-D-galactopyranoside (pNP-β-Gal). Nitrophenolate production was monitored by absorbance at 400 nm; rates were calculated using an extinction coefficient corrected for absorbance at pH 6.5 of 6200 cm^-1^ M^-1^. All experiments were performed in triplicate. Michaelis-Menten parameters were determined by non-linear curve fitting using GraphPad Prism 5.0. The *K*_i_ values for GNJ and GIF were determined from plots of the apparent Km/Vmax against inhibitor concentration. Inhibition experiments were done under the above conditions using 3 nM of enzyme.

**Binding studies.** Qualitative UV difference scans were performed using methods already described (Ficko-Blean and Boraston, 2006, 2009). The concentration of CBM used for the titrations was 36 μM in PBS pH 7.4. The concentration of the carbohydrate solution used to titrate into the protein was ~20 mM and was prepared by mass in PBS pH 7.4. Experiments were performed at 25^o^C in triplicate.

ITC was performed essentially as described previously using a VP-ITC system (MicroCal, Northampton, MA) (Ficko-Blean et al., 2008; Pluvinage et al., 2011). GH2 protein was extensively dialyzed against buffer (100 mM potassium phosphate buffer, pH 6.5). The GIF was prepared by mass to a concentration of 300 µM in buffer saved from the dialysis step. Protein and inhibitor solutions were filtered and degassed immediately prior to use. GH2 protein was used at a concentration of 12.7 µM. Titrations were performed at 37^o^C in triplicate. The data were fit with a single binding site model to determine binding parameters. Similarly, CBM71-1 and CBM71-2 proteins were dialyzed against PBS pH 7.4 and LacNAc and lactose were prepared by mass in buffer saved from dialysis. The CBMs were used at a concentration of 200 µM and carbohydrates at concentrations of 5 mM. Titrations were performed in triplicate at 25^o^C. Due to the relatively low affinity of the interactions and subsequently low C-values [[14](#_ENREF_14)] that were less than 5, the stoichiometry (*n* value) was fixed at one during model fitting. This estimated stoichiometry was in accordance with the 1:1 stoichiometry observed in the crystal structure of CBM71-1.

**Generation** **of** ***S.*** ***pneumoniae*** **mutant**. *S. pneumoniae* TIGR4 Δ*bgaA* strain, was obtained by a PCR ligation technique to replace the *bgaA* gene with a chloramphenicol cassette (Abbott et al., 2010; Lau et al., 2002). Other *S. pneumoniae* mutant strains were generated using the Janus cassette system (Sung et al., 2001). Construction of a mutant strain using this method requires two rounds of transformation. The first round introduces a Janus cassette, which encodes kanamycin resistance and streptomycin sensitivity (*rpsL*^-^), into the genome of a streptomycin resistant *S. pneumoniae* strain in place of the region to be genetically modified. DNA fragments flanking the region to be deleted were amplified (primers 1 and 2 and primers 3 and 4) (Table S2) and sequentially joined to the Janus cassette PCR product (primers J.F and J.R) using a variation of the process of splicing by overlap extension (SOE) by PCR (Burnaugh et al., 2008), first described by Horton et al. [[15](#_ENREF_15)]. All genomic DNA was prepared as previously described [[16](#_ENREF_16)]. In order to minimize PCR-generated errors, all PCRs were conducted using a high-fidelity proofreading polymerase (Phusion, NEB). The Janus construct was transformed into *S. pneumoniae*; the transformants were selected on kanamycin and confirmed by PCR (primers 7 and 8). Janus intermediates were not constructed in this study for generation of all the mutant strains. The R6BgaAC and R6BgaAE564R strains were both constructed using the previously generated R6BgaAE564Q Janus intermediate strain (Limoli et al., 2011). As C06_18 is more difficult to transform, Janus intermediates were constructed by transforming C06_18 Sm^r^ with pDrive containing PCR products amplified from the appropriate R6 intermediates (primers 1X and 4X) and cloned using the InFusion EcoDry cloning kit (Clontech) (pDBgaAE564Q Janus and pDBgaAN Janus).

The second round of transformation replaced the Janus cassette with an engineered segment of DNA, recombination of which resulted in the introduction of the desired mutation. For construction of BgaAN and BgaAC fragments flanking the desired deletion were amplified (primers 1 and 5 and primers 6 and 4) and joined by SOEing (primers 1 and 4). Replacement fragments for C06_18 intermediates were cloned in pDrive (pDBgaAN and pDBgaAC) and plasmids used for transformation. For R6Δ*bgaA SgbgaA^+^* four fragments were joined by SOEing, two fragments that encode *S. gordonii* BgaA (primers 9 and 10 and primers 11 and 12), and two fragments flanking BgaA in R6 (primers 13 and 14 and primers 15 and 16) to allow recombination with the R6 intermediate. For generation of R6BgaAE564R a gene fragment encoding BgaA amino acid residues 146-990 (primers 18 and 19) was cloned into pOPINF to generate pOPINFBgaAN. The QuikChange II site-directed mutagenesis kit (Stratagene, La Jolla, CA) was used to introduce a site specific mutation altering Glu 564 to Arg (primers 20 and 21) to generate pOPINFBgaAE564R. A fragment from this plasmid (primers 22 and 23) was used to transform the R6BgaAE564Q Janus intermediate. For generation of R6BgaAW1514A,W1864A, a fragment of *bgaA* and flanking sequence (primers C.1 and C.4) was cloned into pJET1.2/blunt to generate pJET1.2BgaA. We conducted site-directed mutagenesis of tryptophan residues 1514 and 1864 to alanine (primer pairs 24 and 25, and 26 and 27) to generate pJET1.2BgaAW1514A,W1864A. Plasmid was transformed into R6BgaAN Janus. All final mutants were selected on streptomycin and confirmed by PCR with primers flanking the construct (primers 7 and 8, except R6Δ*bgaA SgbgaA^+^* where primers 16 and 17 were used) and where appropriate activity assays and sequencing.

**β-galactosidase activity**. β-Galactosidase assays were performed using a modification of the colorimetric assay using 2-Nitrophenyl-β-galactoside as a substrate, as described previously (King et al., 2006). Briefly, *S. pneumoniae* were grown in THY to an OD_600_ of 0.6 and cells were harvested by centrifugation at 3000 x g. Cells were resuspended in an equal volume of 0.1M sodium phosphate buffer pH 7.4 and enzyme reactions were performed. Where indicated, β-galactosidase inhibitor (25-2500nM) was added to the reactions. Miller units were calculated as described previously (King et al., 2006). Assays were performed in triplicate on three independent occasions.

**Growth assays.** The protocol for the growth assays was adapted from Battig et al., 2006. Wild-type and Δ*bgaA S. pneumoniae* TIGR4 strains were streaked onto blood agar plates and incubated at 37°C in a candle jar for 12 h. Overnight cultures were set up by inoculating 10 colonies into AGCHY broth supplemented with 1% (w/v) glucose for 9 h. The overnight cultures were used to inoculate fresh AGCHY with 1% (w/v) glucose that were grown until reaching an OD_600_ of 0.5–0.6. Cells were then washed with AGCHY broth and subsequently diluted 1/50 into AGCHY with an additional 1% (w/v) of glucose, bovine asialofetuin (ASF), each in triplicate. Optical densities at 600 nm were read at regular intervals. The half maximal effective concentration (EC_50_) was determined graphically.

**Opsonophagocytic killing assays.** Neutrophil killing assays were performed essentially as previously described (Dalia et al., 2010, Davis et al., 2008). Briefly, 10^3^ PBS-washed late-log-phase bacteria (in 10 µl) were preopsonized in 20 µl of a complement source (10% normal human serum), followed by incubation with 10^5^ neutrophils freshly isolated from human whole blood (40 µl) in 130 µl Hanks balanced salt solution (HBSS) buffer containing Ca^2+^ and Mg^2+^ (GIBCO) plus 0.1% gelatin (+++ buffer). Reaction mixtures were then incubated at 37°C for 45 min with rotation. Opsonophagocytic killing assays were performed with *S. pneumoniae* TIGR4 wild type strain with or without inhibitors and Δ*bgaA* strain in the presence of inhibitors or a vehicle control (+++ buffer). The reactions were stopped by incubation in ice, and viable counts of bacteria were determined by dilution plating. Percent survival was determined relative to control reactions where no neutrophils were added. Statistical significance was assessed using two-tailed *t*-test comparisons.

**Adherence assays.** Adherence *of S. pneumoniae* to mono layer of Detroit 562 cells (D562; ATCC CCL-138), a human pharyngeal carcinoma cell line, and NHBE (Lonza), primary normal human bronchial epithelial cells, grown in 24 well tissue culture plates was determined essentially as described previously (Gould and Weiser, 2002; Limoli et al., 2011). Briefly, *S. pneumoniae* strain were grown in THY medium to an OD_600_ of 0.6 and diluted in antibiotic-free tissue-culture medium. Epithelial cells were pretreated for 30 min with 0.01 U of purified *Clostridium perfringens* sialidase per well and ~1x10^5^ bacteria were added to each well and then allowed to adhere at 37 °C for 60 min. Non-adherent bacteria were removed by five washes in PBS (three washes for NHBE), bacteria adherent to epithelial cells were lifted with 0.25% trypsin/1 mM EDTA and enumerated by serial dilution. All adherence assays were performed in triplicate on at least three independent occasions. The percentage adherence of a strain was calculated as the proportion of the inoculum that was adherent to the epithelial cells. Adherence of the parental strains was adjusted to 100 % and the adherence of the mutants was expressed relative to that of the parental strain under the same experimental conditions. When testing the effects of molecules on adherence, adherence was expressed relative to that of the same strain in the absence of the molecule. Data are presented as means ± SD. Statistical significance was determined using two tailed Student’s *t*-test and data points with *p* value ≤ 0.05 were considered significant. Trypan blue staining was performed to ensure that the molecules added to adherence assays were not toxic to the epithelial cells at the concentrations used. Epithelial cells were incubated with the highest dose of additive used for 60 min, washed and lifted with trypsin as described above. Subsequently, 25 µl cells was added to an equal volume of 0.4 % trypan blue and cells were counted using TC-10 automated cell counter (Bio-Rad).

**Cell adhesion assay to immobilized CBM.** To determine if CBMs directly bound to host cells we measured the ability of D562 cells to bind to immobilized CBMs. CBM71-1 and CBM71-2 were diluted in PBS pH 7.4 at a range of concentrations (2.5-20.0 µg ml^-1^) and 100 µl used to coat wells of a 96-well plate overnight at 4 ^o^C. Control wells were coated with 1% BSA (w/v) in PBS. Prior to binding assays wells were blocked with 1% BSA in PBS for 1.5 h at room temperature and washed 3 times with PBS. D562 cells grown in 75 cm^2^ tissue culture flasks to 70-80% confluency were lifted with 2 ml of 10 mM EDTA in PBS. Cells were collected by centrifugation at 230 x g for 5 min at room temperature, washed with 4 ml of MEM with 2% FBS and resuspended in 9 ml of MEM with 2% FBS. Cells were treated with 0.15 µM of *C. perfringens* sialidase (*Cp*Sia) or sialidase and 0.054 µM *S. pneumoniae* BgaA146-990 for 30 min at 37 ^o^C, immediately prior to the assay. 5x10^4^ D562 cells were added to each well and allowed to adhere to the plate for 1 h at 37 ^o^C. Unbound cells were removed by three washes with MEM and cells were fixed with 50 µl of 2.5% gluteraldehyde (w/v) for 30 min at room temperature. Wells were washed with distilled water and cells were stained with 50 µl of 0.1% crystal violet (w/v) for 30 min. Following five washes with distilled water adherent cells were counted using an inverted light microscope. The average number of cells bound to BSA coated wells was subtracted from the number of cells attached to each CBM coated well. Three independent experiments were performed to calculate the average ± SD. Statistically significant differences between binding of D562 cells treated with sialidase and sialidase and β-galactosidase was determined using two tailed Student’s *t*-test and data points with *p* value ≤ 0.05 were considered significant.

**Sequence alignments.** Alignments were generated using ClustalW2 (<http://www.ebi.ac.uk/Tools/msa/clustalw2/>) [[17](#_ENREF_17)] and shading performed using ShadeBox (<http://embnet.vital-it.ch/software/BOX_form.html> )

**SUPPORTING REFERENCES**

1. Ichikawa H, Igarashi Y, Ichikawa M, Suhara Y (1998) 1-*N-*Iminosugars: potent and selective inhibitors of b-glycosidases. J Am Chem Soc 120: 3007-3018.

2. Barik S (1996) Site-directed mutagenesis in vitro by megaprimer PCR. Methods Mol Biol 57: 203-215.

3. McCoy AJ, Grosse-Kunstleve RW, Adams PD, Winn MD, Storoni LC, et al. (2007) Phaser crystallographic software. J Appl Crystallogr 40: 658-674.

4. Cowtan K (2006) The Buccaneer software for automated model building. 1. Tracing protein chains. Acta Crystallogr D Biol Crystallogr 62: 1002-1011.

5. Emsley P, Cowtan K (2004) Coot: model-building tools for molecular graphics. Acta Crystallogr D Biol Crystallogr 60: 2126-2132.

6. Murshudov GN, Vagin AA, Dodson EJ (1997) Refinement of macromolecular structures by the maximum-likelihood method. Acta Crystallogr D Biol Crystallogr 53: 240-255.

7. Cowtan K, Main P (1998) Miscellaneous algorithms for density modification. Acta Crystallogr D Biol Crystallogr 54: 487-493.

8. Morris RJ, Perrakis A, Lamzin VS (2002) ARP/wARP's model-building algorithms. I. The main chain. Acta Crystallogr D Biol Crystallogr 58: 968-975.

9. Brunger AT (1992) Free R value: a novel statistical quantity for assessing the accuracy of crystal structures. Nature 355: 472-475.

10. Vaguine AA, Richelle J, Wodak SJ (1999) SFCHECK: a unified set of procedures for evaluating the quality of macromolecular structure-factor data and their agreement with the atomic model. Acta Crystallogr D Biol Crystallogr 55: 191-205.

11. Laskowski RA, Moss DS, Thornton JM (1993) Main-chain bond lengths and bond angles in protein structures. J Mol Biol 231: 1049-1067.

12. Davis IW, Leaver-Fay A, Chen VB, Block JN, Kapral GJ, et al. (2007) MolProbity: all-atom contacts and structure validation for proteins and nucleic acids. Nucleic Acids Res 35: W375-383.

13. Chen VB, Arendall WB, 3rd, Headd JJ, Keedy DA, Immormino RM, et al. (2010) MolProbity: all-atom structure validation for macromolecular crystallography. Acta Crystallogr D Biol Crystallogr 66: 12-21.

14. Wiseman T, Williston S, Brandts JF, Lin LN (1989) Rapid measurement of binding constants and heats of binding using a new titration calorimeter. Anal Biochem 179: 131-137.

15. Horton RM, Hunt HD, Ho SN, Pullen JK, Pease LR (1989) Engineering hybrid genes without the use of restriction enzymes: gene splicing by overlap extension. Gene 77: 61-68.

16. Whatmore AM, Barcus VA, Dowson CG (1999) Genetic diversity of the streptococcal competence (*com*) gene locus. J Bacteriol 181: 3144-3154.

17. Larkin MA, Blackshields G, Brown NP, Chenna R, McGettigan PA, et al. (2007) Clustal W and Clustal X version 2.0. Bioinformatics 23: 2947-2948.
