# Supplementary material for: Unravelling the Multiple Functions of the Architecturally Intricate Streptococcus pneumoniae β-galactosidase, BgaA
Source: PLoS Pathog. 2014 Sep 11;10(9):e1004364. doi: 10.1371/journal.ppat.1004364 (PMC4161441; doi:10.1371/journal.ppat.1004364)
Supplement: Table S1 — Bacterial strains and plasmids used in the study. (DOCX) [file ppat.1004364.s009.docx]

**Table S1: Bacterial strains and plasmids used in the study**

| **Bacterial strain/plasmid** | **Genotype/Characteristics^a^** | **Source/Reference** |
| --- | --- | --- |
| S. pneumoniae | | |
| R6 | Laboratory strain | Iannelli et al., 1999 |
| R6ΔbgaA | ΔbgaA (Em^r^) | King et al., 2006 |
| R6Sm^r^ | *rpsL*K56T (Sm^r^) | Limoli et al., 2011 |
| R6BgaAE564Q Janus | Δ*bgaA* (Km^r^, Sm^s^) | Limoli et al., 2011 |
| R6BgaAE564R | Gln564→Arg in BgaA, *rpsL*K56T (Sm^r^) | This study |
| R6BgaAC | BgaA∆47-991, *rpsL*K56T (Sm^r^) | This study |
| R6BgaAN Janus | Δ*bgaA* (Km^r^, Sm^s^) | This study |
| R6BgaAN | BgaA∆991-1984, *rpsL*K56T (Sm^r^) | This study |
| C06_18 | Clinical isolate from blood | Burnaugh et al., 2008 |
| C06_18 Sm^r^ | *rpsL*K56T (Sm^r^) | This study |
| C06_18Δ*bgaA* | Δ*bgaA* (Em^r^) | Limoli et al., 2011 |
| C06_18BgaAE564Q Janus | Δ*bgaA* (Km^r^, Sm^s^) | This study |
| C06_18BgaAC | BgaA∆47-991, *rpsL*K56T (Sm^r^) | This study |
| C06_18BgaAN Janus | Δ*bgaA* (Km^r^, Sm^s^) | This study |
| C06_18BgaAN | BgaA∆991-1984, *rpsL*K56T (Sm^r^) | This study |
| TIGR4 | ATCC BAA-334/Clinical isolate from blood | Tettelin et al., 2001 |
| TIGR4∆*bgaA* | ∆*bgaA* (Cam^r^) | This study |
| *Escherichia* *coli* | | |
| *E. coli* Stellar^TM^ | *F*–*, ara,Δ(lac-proAB) [*Φ*80d lacZΔM15], rpsL(str), thi, Δ(mrr-hsdRMS-mcrBC), ΔmcrA, dam, dcm* | Clontech |
| *E. coli* BL21 Star | F^-^*ompT hsdS*_B_(r_B_^-^ m_B_^-^) *gal dcm*(DE3) pRARE (Cam^r^) | Invitrogen |
| Plasmids | | |
| pET28b | T7 based expression vector, Km^r^ | Novagen |
| pETGH2 | pET28b encodes BgaA GH2, Km^r^ | This study |
| pETBgaACBM1 | pET28b encodes *Sp*BgaACBM71-1, Km^r^ | This study |
| pETBgaACBM2 | pET28b encodes *Sp*BgaACBM71-2 Km^r^ | This study |
| pETBgaACBM1-2 | pET28b encodes *Sp*BgaACBM71-1.2, Km^r^ | This study |
| pOPINF | T7 based expression vector, Amp^r^ | Berrow et al., 2007 |
| pOPINFBgaAN | pOPINF construct to generate pOPINFBgaAE564R, Amp^r^ | This study |
| pOPINFBgaAE564R | pOPINF construct to generate R6BgaAE564R strain, Amp^r^ | This study |
| pDrive | PCR cloning vector, Amp^r^ Km^r^ | Qiagen |
| pDBgaAE564Q Janus | pDrive construct to generate C06_18E564Q Janus strain, Amp^r^ Km^r^ | This study |
| pDBgaAC | pDrive construct to generate C06_18BgaAC strain, Amp^r^ Km^r^ | This study |
| pDBgaAN Janus | pDrive construct to generate C06_18BgaAN Janus strain, Amp^r^ Km^r^ | This study |
| pDBgaAN | pDrive construct to generate C06_18BgaAN strain, Amp^r^ Km^r^ | This study |
| pJET1.2/blunt | Blunt end cloning vector, Amp^r^ | Thermo Scientific |
| pJET1.2BgaA | pJET1.2/blunt construct to generate pJET1.2BgaAW1514A,W1864A | This study |
| pJET1.2BgaAW1514A,W1864A | pJET1.2/blunt construct to generate R6BgaAW1514A,W1864A | This study |

^a^ Em^r^ resistant to erythromycin, Km^r^ resistant to kanamycin, Sm^r^ resistant to streptomycin, Sm^s^ sensitive to streptomycin, Cam^r^ resistant to chloramphenicol, Amp^r^ resistant to ampicillin.
