# Supplementary material for: Unravelling the Multiple Functions of the Architecturally Intricate Streptococcus pneumoniae β-galactosidase, BgaA
Source: PLoS Pathog. 2014 Sep 11;10(9):e1004364. doi: 10.1371/journal.ppat.1004364 (PMC4161441; doi:10.1371/journal.ppat.1004364)
Supplement: Table S2 — Primers used in the study. (DOCX) [file ppat.1004364.s010.docx]

**Table S2: Primers used in the study**

| **Group** | **Primer** | **Sequence (5’-3’)** | **Location** |
| --- | --- | --- | --- |
| BgaA110-985 |  | CATATGGCTAGCTCTGCTAAACCGGAAGAAAAAGCTCCAAGG | 615769-615798^1^ |
|  |  | GAATTCGCGGCCGCTTATTCTTTCTTACCAGTAAAGACAGTG | 618369-618393^1^ |
| R6BgaAE564R | M 18 | AAGTTCTGTTTCAGGGCCCGATGATTGAAGACAGGAAAGTGG^a^ | 572558-572579^m^ |
|  | M 19 | ATGGTCTAGAAAGCTTTACAAAACAGTCTTCTCTTGTC^a^ | 575095-575076^m^ |
|  | M 20 | CCCTGCTATCTTCATGTGGTCAATTGGTAATAGAATAGGTGAAGCTAATGG | 573781-573831^m^ |
|  | M 21 | CCATTAGCTTCACCTATTCTATTACCAATTGACCACATGAAGATAGCAGGG | 573781-573831^m^ |
|  | M 22 | TGGCGTCTACATGGATTCTCAAGT | 572824-572847^m^ |
|  | M 23 | ACGGGAGTTTGATTTTGGTTGTG | 574305-574283^m^ |
|  | M 7 | TTATGCGGATGAAACACTTAT | 572251-572271^m^ |
|  | M 8 | AGCACGAACTGGAATCTTACCT | 574492-574471^m^ |
| BgaAC | C 1 | CTTTCAGGTACACTTATTGCACC | 571543-571565^m^ |
|  | C 5 | CAAGTGATGATGGTGGTGATGATCCGCATAAACTACAGATTCTC^b^ | 572260-572238^m^ |
|  | C 6 | GATCATCACCACCATCATCACTTGGGGACAGAGGTGCCAAAAGT^c^ | 575093-575115^m^ |
|  | C 4 | GGCTGAATAAAGATGCTCGCACGC | 575733-575710^m^ |
|  | C 7 | GTATCATCGCTATGGGTGTTAC | 571442-571463^m^ |
|  | C 8 | TTGTAGAGAAGGTTACTTTATC | 575927-575906^m^ |
|  | C 1X | TCGGATCCAGAATTCCTTTCAGGTACACTTATTGCACC^d^ | 571543-571565^m^ |
|  | C 4X | CTTGTCGACGAATTCGGCTGAATAAAGATGCTCGCACGC^d^ | 575733-575710^m^ |
| BgaAN | N 1 | GGTACGGACTATATTGGTGAAC | 574250-574271^m^ |
|  | N 2 | CATTATCCATTAAAAATCAAACGGTGCCGCTGGCTTACCAGCAG^e^ | 574729-574710^m^ |
|  | N 3 | AAGCATAAGGAAAGGGGCCCCAAACTAATAAAGCCCAGTTACC^f^ | 578684-578706^m^ |
|  | N 4 | GTTTCAATCTACTATACAATAAGAG | 578983-578959^m^ |
|  | N 5 | CTGCTGGTAAGCCAGCGGCACAAACTAATAAAGCCCAGTTACC^g^ | 578684-578706^m^ |
|  | N 6 | TGCCGCTGGCTTACCAGCAG | 574729-574710^m^ |
|  | N 7 | CCGTGACAACGCTGGCTATGCTG | 574210-574232^m^ |
|  | N 8 | CTCCTGAAGGTAGACAGATTTCGCT | 579234-579210^m^ |
|  | N 1X | TCGGATCCAGAATTCGGTACGGACTATATTGGTGAAC^d^ | 574250-574271^m^ |
|  | N 4X | CTTGTCGACGAATTCGTTTCAATCTACTATACAATAAGAG^d^ | 578983-578959^m^ |
| R6BgaAW1514A,W1864A | W 24 | CAGCCAATCGTTGGACAAACGCGAATCGTAGTAATCCAGAAG | 576642- 576683^m^ |
|  | W 25 | CTTCTGGATTACTACGATTCGCGTTTGTCCAACGATTGGCTG | 576642- 576683^m^ |
|  | W 26 | AAATCGTCGTTGGACAAACGCGTCACCAACACCATCTTCT | 577693- 577732^m^ |
|  | W 27 | AGAAGATGGTGTTGGTGACGCGTTTGTCCAACGACGATTT | 577693- 577732^m^ |
| Janus | JF | CCGTTTGATTTTTAATGGATAATG | 7-30^n^ |
|  | JR | GGGCCCCTTTCCTTATGCTT | 247511-247527^l^ |
| R6Δ*bgaA SgbgaA*^+^ | G 1 | ATTCGTAAGTTTGCTGTGGGAG | 572162-572183^m^ |
|  | G 2 | CATTATCCATTAAAAATCAAACGTTTAGCATCTTCCTTGAGCGG^e^ | 572530-572510^m^ |
|  | G 3 | AAGCATAAGGAAAGGGGCCCTACAAGAAGATCTTCCAAAACTC^f^ | 578379- 578401^m^ |
|  | G 4 | GGTCTTTAATGATAAAGAAGGTAT | 578841-578818^m^ |
|  | G 7 | AAGGCCATTGGAATCGG | 572130-572146^m^ |
|  | G 8 | TATTTAGGACAAGAGTTTTTC | 578865-578845^m^ |
|  | G 9 | ATGGAAAAAGGATATTGGAATC | 1543009-1542988^o^ |
|  | G 10 | CATAGGTCTGTAGGGTACCGCC | 1539370-1539391^o^ |
|  | G 11 | TAGTCATTCAGGCATTGTAACC | 1535957-1535978^o^ |
|  | G 12 | TCAGTCTTCTTTTCTACGTTTC | 1535957-1535978^o^ |
|  | G 13 | CTTTCAGGTACACTTATTGCACC | 572122-572098^m^ |
|  | G 14 | GATTCCAATATCCTTTTTCCATAAAACCCTCCTTATATTATATTTAG^h^ | 572122-572098^m^ |
|  | G 15 | GAAACGTAGAAAAGAAGACTGAAAATTTTGATACCTTCTTTATC^i^ | 578810-578832^m^ |
|  | G 16 | CTCCTGAAGGTAGACAGATTTCGCT | 579234-579210^m^ |
|  | G 17 | GTATCATCGCTATGGGTGTTAC | 571442-571463^m^ |
| CBM | CBM71-1F | CGCGCGGCAGCCATATGCAAACTGAGCAAGGTGCAA^j^ | 576509-576527^m^ |
|  | CBM71-1R | GACGGAGCTCGAATTCTTAAACTTGTTTCGCAAAGATT^k^ | 577057-577039^m^ |
|  | CBM71-2F | CGCGCGGCAGCCATATGGCTAGCATGAAACTTGGTGAGACTC^j^ | 577559-577574^m^ |
|  | CBM71-2R | GACGGAGCTCGAATTCTTATTCACTTGGTGCAAGGA^k^ | 578117-578100^m^ |

^a^Underlining indicates nucleotides introduced to allow In-Fusion^TM^ cloning with the pOPINF vector, Berrow et al., 2007

^b^Underlining indicates nucleotides introduced to encode 6xHis tag

^c^Underlining indicates reverse complement of ^b^

^d^Underlining indicates nucleotides introduced to allow In-Fusion^TM^ cloning with pDrive

^e^Underlining indicates the reverse complement sequence of primer JF

^f^Underlining indicates the reverse complement sequence of primer JR

^g^Underlining indicates the reverse complement sequence of primer N6

^h^Underlining indicates the reverse complement sequence of primer G9

^i^Underlining indicates the reverse complement sequence of primer G12

^j^Underlining indicates nucleotides introduced to allow In-Fusion^TM^ cloning with pET28b, nucleotides 225-241

^k^Underlining indicates nucleotides introduced to allow In-Fusion^TM^ cloning with pET28b, nucleotides 182-196

^l^NCBI accession number AE005672

^m^NCBI accession number AE007317

^n^NCBI accession number AY334019

^o^NCBI accession number CP000725
