# Supplementary material for: Unravelling the Multiple Functions of the Architecturally Intricate Streptococcus pneumoniae β-galactosidase, BgaA
Source: PLoS Pathog. 2014 Sep 11;10(9):e1004364. doi: 10.1371/journal.ppat.1004364 (PMC4161441; doi:10.1371/journal.ppat.1004364)
Supplement: Table S3 — X-ray data collection and structure statistics. Values in parentheses are for the highest resolution bin. (DOCX) [file ppat.1004364.s011.docx]

**Table S3: X-ray data collection and structure statistics. Values in parentheses are for the highest resolution bin.**

|  | BgaA catalytic domain | | | BgaA catalytic domain E645Q | CBM71-1 | | CBM71-2 |
| --- | --- | --- | --- | --- | --- | --- | --- |
|  | Native | GIF complex | GNJ complex | LacNAc complex | Seleno-methionine | LacNAc complex | Native |
| ***Data Collection*** |  |  |  |  |  |  |  |
| Beamline | MM-003 | SSRL BL9-2 | SSRL BL9-2 | CLS 08ID-1 | CLS 08BM-1 | CLS 08BM-1 | CLS 08BM-1 |
| Wavelength (Å) | 1.5418 | 0.97946 | 0.97911 | 0.97949 | 0.97874 | 0.97874 | 0.97874 |
| Space Group | *P*4_3_ 2 2 | *P*4_3_ 2 2 | *P*4_3_ 2 2 | *P*4_3_ 2 2 | *P*4_3_ 2_1_ 2 | *P*4_3_ 2_1_ 2 | *P*2_1_ 2_1_ 2_1_ |
| Cell Dimensions |  |  |  |  |  |  |  |
| *a, b, c* (Å) | 116.8, 116.8, 220.0 | 116.8, 116.8, 219.7 | 116.6, 116.6, 219.5 | 116.5, 116.5, 218.4 | 70.3, 70.3, 159.7 | 70.5, 70.5, 161.6 | 38.3, 69.3, 121.2 |
| Resolution (Å) | 19.94-2.70 (2.80-2.70) | 38.93-2.20 (2.25-2.20) | 40.0-2.50 (2.64-2.50) | 52.49-2.20 (2.32-2.20) | 36.33-1.54 (1.63-1.54) | 40.00-2.10 (2.21-2.10) | 45.61-1.83 (1.93-1.83) |
| R_merge_ | 0.113 (0.425) | 0.094 (0.210) | 0.132 (0.438) | 0.197 (0.638) | 0.079 (0.396) | 0.153 (0.408) | 0.098 (0.309) |
| <I/σI> | 10.9 (3.3) | 14.2 (6.7) | 16.2 (6.7) | 10.1 (5.6) | 21.9 (7.0) | 9.5 (4.7) | 13.3 (6.4) |
| Completeness (%) | 99.7 (100) | 99.6 (93.7) | 100 (100) | 100 (100) | 100 (100) | 100 (100) | 99.8 (100) |
| Redundancy | 4.6 (4.7) | 7.3 (5.9) | 11.5 (11.7) | 9.8 (9.8) | 16.6 (16.5) | 9.0 (9.3) | 7.5 (7.5) |
| No. of reflections | 195851 | 564790 | 612584 | 767439 | 994164 | 222871 | 220349 |
| No. Unique | 42431 | 77385 | 53140 | 78624 | 59949 | 24686 | 29320 |
|  |  |  |  |  |  |  |  |
| ***Refinement*** |  |  |  |  |  |  |  |
| Resolution (Å) | 2.70 | 2.20 | 2.50 | 2.20 | 1.54 | 2.10 | 1.83 |
| R_work_/R_free_ | 0.18/0.22 | 0.15/0.18 | 0.16/0.21 | 0.15/0.19 | 0.16/0.20 | 0.19/0.25 | 0.17/0.22 |
| No. of atoms |  |  |  |  |  |  |  |
| Protein | 6815 | 7072 | 6909 | 7015 | 1457 (A), 1449 (B) | 1420 (A), 1433 (B) | 1378 (A), 1460 (B) |
| Ligand | 156 (EDO), 25 (SO4) | 10 (GIF), 40 (SO4), 100 (EDO) | 11 (GNJ), 200 (EDO),  25 (SO4) | 26 (LacNAc), 30 (SO4),  40 (MPD) | 44 (EDO), 2 (CA) | 26 (LacNAc A), 26 (LacNAc B), 40 (EDO), 2 (CA) | 20 (EDO), 2 (CA) |
| Water | 492 | 982 | 644 | 800 | 702 | 307 | 248 |
| *B*-factors |  |  |  |  |  |  |  |
| Protein | 24.1 | 19.7 | 23.7 | 22.2 | 14.1 (A), 14.4 (B) | 20.2 (A), 22.3 (B) | 16.0 (A), 18.3 (B) |
| Ligand | 33.8 (EDO), 39.2 (SO4) | 12.6 (GIF), 40.4 (SO4), 40.5 (EDO) | 17.2 (GNJ), 46.8 (EDO), 47.9 (SO4) | 30.3 (LacNAc), 52.5 (SO4), 55.6 (MPD) | 23.0 (EDO), 8.34 (CA) | 30.9 (LacNAc A), 24.9 (LacNAc B), 29.7 (EDO), 17.5 (CA) | 27.7 (EDO), 12.2 (CA) |
| Water | 23.8 | 31.5 | 27.0 | 33.0 | 31.2 | 30.3 | 25.1 |
| r.m.s.d |  |  |  |  |  |  |  |
| Bond lengths (Å) | 0.011 | 0.011 | 0.011 | 0.011 | 0.018 | 0.010 | 0.013 |
| Bond angles (°) | 1.416 | 1.385 | 1.361 | 1.452 | 1.873 | 1.363 | 1.479 |
| Ramachandran (%) |  |  |  |  |  |  |  |
| Preferred | 98.1 | 98.1 | 96.7 | 97.8 | 98.9 | 98.1 | 95.2 |
| Allowed | 1.5 | 1.7 | 3.0 | 2.0 | 1.1 | 1.9 | 4.8 |
| Disallowed | 0.4 | 0.2 | 0.3 | 0.2 | 0.0 | 0.0 | 0.0 |
