# Supplementary material for: Unravelling the Multiple Functions of the Architecturally Intricate Streptococcus pneumoniae β-galactosidase, BgaA
Source: PLoS Pathog. 2014 Sep 11;10(9):e1004364. doi: 10.1371/journal.ppat.1004364 (PMC4161441; doi:10.1371/journal.ppat.1004364)
Supplement: Supplementary file 10 [file ppat.1004364.s004.docx]

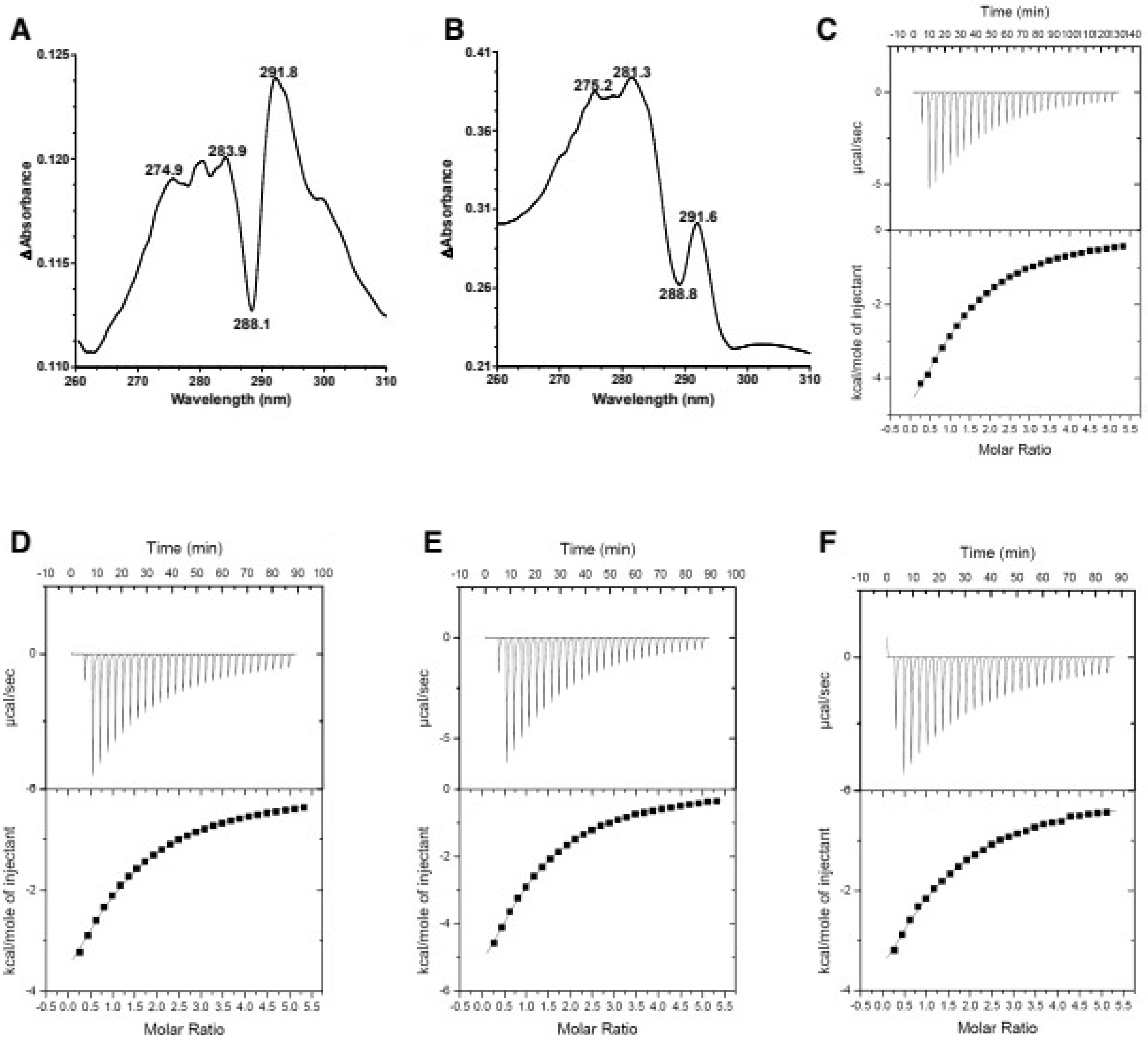
A
ΔAbsorbance
Wavelength (nm)
274.9
283.9
291.8
288.1
B
ΔAbsorbance
Wavelength (nm)
275.2
281.3
291.6
288.8
C
Time (min)
µcal/sec
kcal/mole of injectant
Molar Ratio
D
Time (min)
µcal/sec
kcal/mole of injectant
Molar Ratio
E
Time (min)
µcal/sec
kcal/mole of injectant
Molar Ratio
F
Time (min)
µcal/sec
kcal/mole of injectant
Molar Ratio
